# Autonomous High‐Throughput Characterization of Liquid‐Liquid Phase Behavior

**DOI:** 10.1002/advs.76294

**Published:** 2026-06-30

**Authors:** Tarek Eid, Maryam Ebrahimiazar, Mohammad Zargartalebi, David Sinton

**Affiliations:** ^1^ Department of Mechanical and Industrial Engineering University of Toronto Toronto Ontario Canada

**Keywords:** formulation design, high‐throughput screening, liquid‐liquid equilibrium, miscibility, multimodal sensor, phase behavior, self‐driving labs

## Abstract

Self‐driving labs and data‐driven formulation have outpaced the characterization of liquid‐liquid miscibility and phase behavior, despite the role of such characterization in determining the stability and efficacy of complex formulations across diverse applications. Traditional characterization methods rely on labor‐intensive visual inspection or single‐proxy measurements that lack chemical generality, limit throughput, and provide only partial insight into phase behavior. Here, we report an automated platform that enables continuous, high‐throughput screening of liquid‐liquid phase behavior across diverse fluid chemistries. The device integrates an asymmetric capacitance sensor that is sensitive to the emergence and motion of phase boundaries, along with multi‐angle turbidimetry that quantifies cloudiness and emulsion stability, in a single flow‐through chamber. We demonstrate the classification of chemically diverse binary mixtures, resolution of real‐time phase separation kinetics, and identification of partial miscibility across compositions and temperatures. For multicomponent systems, we employ Gaussian‐process‐based active learning to autonomously map ternary phase diagrams in ∼2 h, together with a nonlinear programming framework that extracts tie lines in ∼5 min per line. By unifying miscibility classification, kinetic characterization, and thermodynamic mapping in a single automated workflow, the platform enables comprehensive phase behavior screening at the scale and throughput required for autonomous formulation discovery.

## Graphical abstract

This study introduces an automated dual modality platform, combining asymmetric capacitance deviation and multiangle turbidimetry, for high‐throughput characterization of liquid‐liquid phase behavior across chemically diverse fluid systems. The platform enables miscibility classification, resolution of phase separation kinetics and emulsion stability, ternary phase diagram mapping, and tie line extraction, addressing the pace and scope required by self‐driving formulation laboratories.

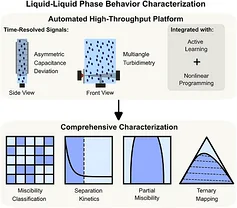

## 1 Introduction

**FIGURE 1:**
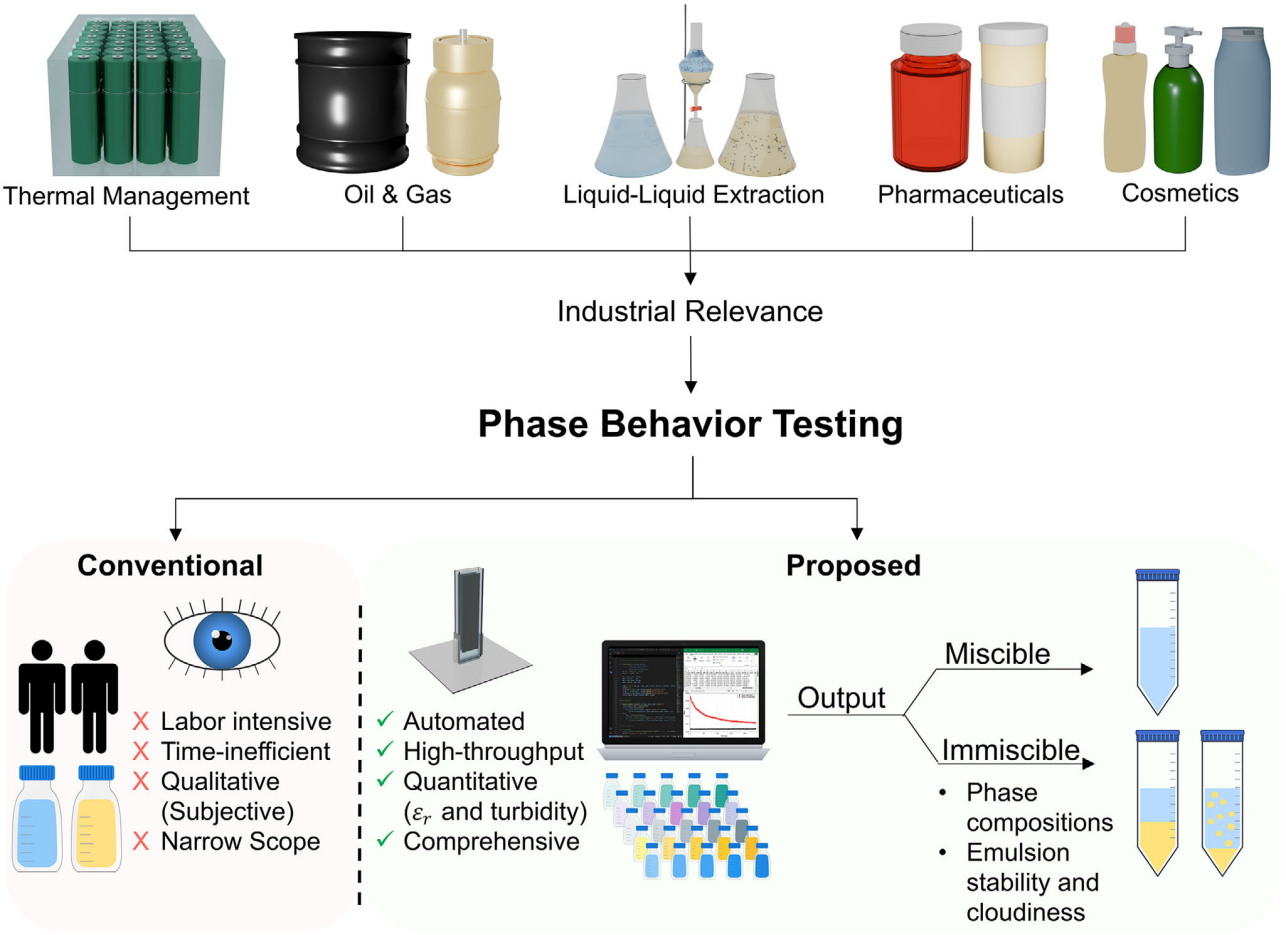
Industrial formulation development demands rapid, chemically generalizable phase behavior characterization to meet application‐dependent phase behavior requirements.

Liquid‐liquid phase behavior is a thermodynamic constraint that governs the feasibility and design of fluid systems ranging from solvent extraction to the development of stable, high‐performance formulations (Figure 1). In many of these processes, the working fluids — such as coolants, lubricants, drilling fluids, and complex blends used in pharmaceutical and cosmetic processing — are typically composed of a multicomponent base along with functional additives [1, 2]. Small changes in component compositions, operating temperature, or the addition of a new component can lead to immiscibility, manifesting as phase separation or cloudiness [3, 4]. Depending on the application, immiscibility must either be prevented to maintain homogeneity and efficacy or deliberately exploited to enable controlled separations [5, 6, 7, 8]. Consequently, it is necessary to determine whether the fluid system of interest is miscible or immiscible, and if immiscible, whether it will form a stable emulsion or undergo droplet coalescence and bulk phase separation [9, 10]. With the discovery of new materials and the rise of self‐driving labs and machine‐learning‐guided tools, large formulation spaces can now be explored rapidly, but phase behavior characterization has not kept pace and remains a bottleneck in accelerated workflows [11, 12, 13].

Phase behavior testing remains dominated by optical techniques, originating from traditional visual inspection and cloud‐point titrations that detect the onset of turbidity [14, 15, 16, 17]. While these methods provide straightforward identification of immiscibility, they are inherently sequential and were developed for manual, low‐throughput studies, typically yielding only a binary miscibility classification at a single condition [18]. Scaling to high‐throughput screening requires direct evaluation of mixed compositions rather than stepwise titrations, but direct optical classification depends on threshold selection that does not generalize across chemically diverse systems and relies on refractive‐index contrast [19, 20, 21]. Without a complementary, non‐optical metric to validate classification, automated high‐throughput miscibility screening based solely on optical signals remains unreliable. Recent efforts have therefore focused on narrow, well‐defined tasks on strong optical contrast systems rather than general miscibility screening. Robotics, droplet‐based workflows, and computer vision methods typically target visible phase separation or partition coefficients, avoiding the need for universal thresholds yet leaving miscibility boundaries unresolved [22, 23, 24, 25, 26, 27]. Raman spectroscopy and interface‐tracking optimization workflows accelerate tie line determination but still require prior binodal knowledge, with determination times of 20–50 min per tie line [28, 29, 30]. Consequently, despite these advances, optical methods still fail to provide a chemically general, autonomous, and comprehensive characterization of miscibility and phase behavior, instead offering only partial information.

Predictive strategies based on dielectric constant and thermodynamic equilibrium modeling offer alternative routes to miscibility assessment without requiring physical measurements. In empirical studies, the dielectric constant, or polarity, has been used as a proxy for miscibility within “like‐dissolves‐like” frameworks by correlating pure‐component polarity with miscibility classifications [31, 32]. Such approaches require reliable dielectric data and assumed mixing rules, limiting applicability to well‐characterized systems and reducing transferability across diverse or strongly associating liquids, with systematic deviations reported for water and other hydrogen‐bonded systems. Even when successful, these correlations provide only a binary miscible/immiscible label without insight into phase separation kinetics, emulsion stability, or tie line compositions. Similarly, high‐throughput thermodynamic frameworks such as the Conductor‐like Screening Model for Real Solvents (COSMO‐RS) and its segment activity coefficient variant (COSMO‐SAC) enable equilibrium and partition coefficient predictions [33, 34]. However, prediction accuracy remains insufficient for polar and complex systems, limiting their reliability as standalone screening tools. Thus, while dielectric‐based and computational approaches provide useful screening heuristics within restricted chemical domains, they lack experimentally grounded, time‐resolved observables, leaving key aspects of phase behavior unmeasured. As a result, they cannot, on their own, support self‐driving labs that must evaluate large numbers of fluid combinations in regions where the required pure‐component properties and model parameters are sparse or unavailable [35, 36, 37].

In this work, we introduce an automated, high‐throughput, and continuous experimental approach that combines post‐mixing capacitance deviation, reflecting dielectric constant changes, and turbidity measurements, quantifying optical clarity, to characterize miscibility and phase behavior. Although capacitance and dielectric constant are directly related through device geometry, it is the post‐mixing deviation in capacitance, rather than its absolute value, that provides generalizable signals independent of pure‐component data or mixing rules. The dual‐modality framework allows turbidity to apply conservative thresholds for rapid and reliable direct evaluation. Together, this asymmetric multimodal approach overcomes the generality, throughput, and blind‐spot limitations of single‐proxy methods. It supports miscibility classification, time‐resolved phase separation analysis, and binodal characterization across composition and temperature; when coupled with Gaussian‐process classification (GPC) and nonlinear programming (NLP), it also enables autonomous mapping of ternary miscibility gaps and tie lines.

## 2 Methods

### 2.1 Chamber Design

**FIGURE 2:**
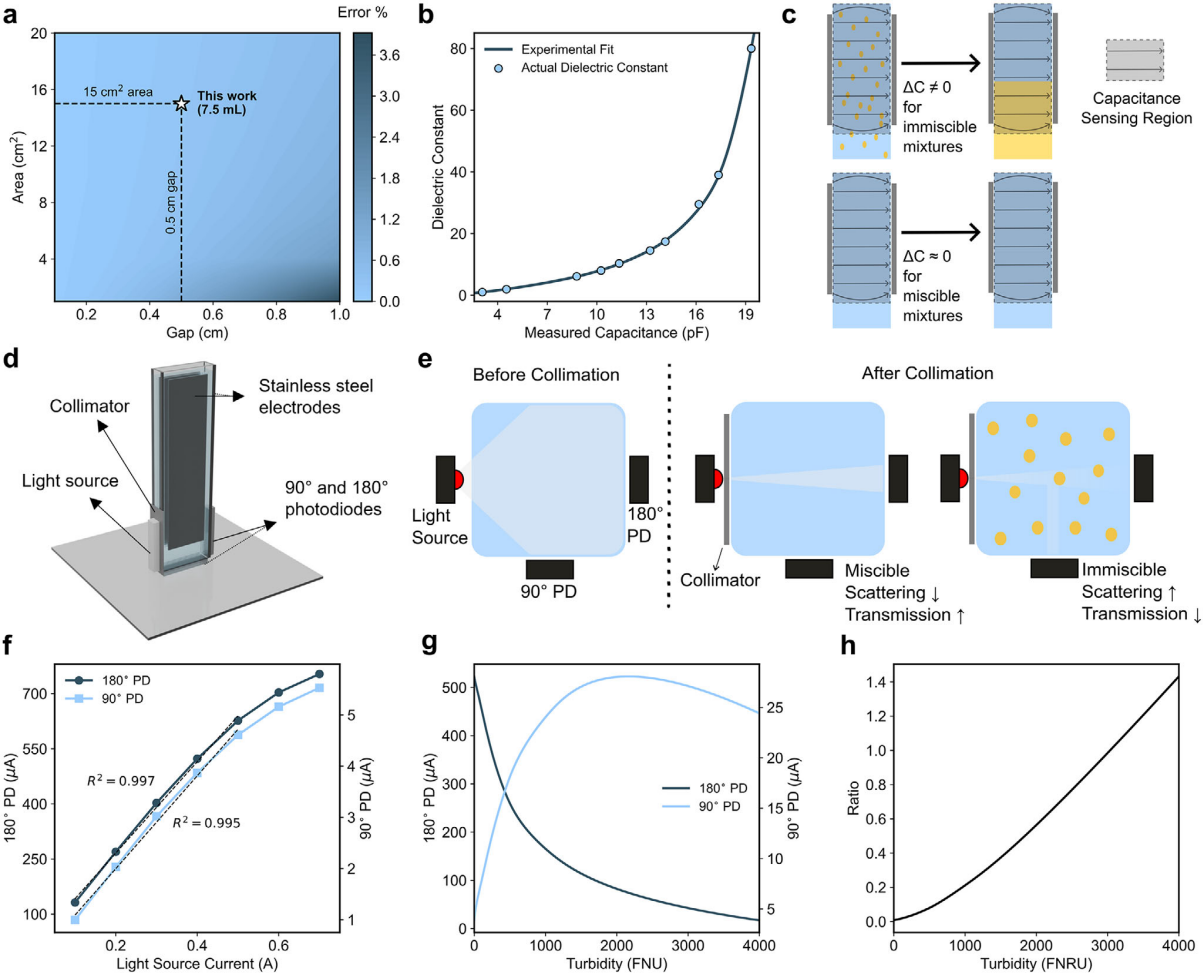
Asymmetric capacitance‐turbidity sensor configuration and calibration. (a) Simulated capacitance error as a function of electrode area and gap spacing. The star denotes the selected 7.5 mL cell geometry, with dashed lines indicating the corresponding gap and chamber area (0.5 cm and 15 cm^2^, respectively). (b) Measured capacitance and corresponding dielectric constant for air and nine reference liquids (heptane, ethyl acetate, 1‐decanol, 1‐octanol, 1‐pentanol, 1‐butanol, propylene glycol, ethylene glycol, and water), ordered left to right by increasing dielectric constant. (c) Post‐mixing capacitance deviation response: immiscible mixtures exhibit ΔC≠0 as phases separate, while miscible mixtures maintain Δ*C*  ≈  0. (d) 3D model of the sensor assembly with collimator, stainless steel electrodes, light source, and 90°/180° photodiodes. (e) Effect of collimation on 90° scattering and 180° transmission paths. (f) Photodiode currents at 90° and 180° versus light source current, showing a linear response from 0.1 to 0.5 A and a collimation‐induced decrease in signal at 90° relative to 180°. (g) 90° and 180° photodiode signals as a function of turbidity, illustrating loss of monotonicity at 90° and reduced sensitivity at 180° for high turbidity. (h) Corresponding scattering‐to‐transmission (90°/180°) ratio, providing an approximately linear optical metric for turbidity classification for the full range.

The sensing chamber consists of rectangular glass pieces, two stainless steel electrodes, two photodiodes, a light source, a collimator, and black tape. The size of the chamber was based upon multiple factors, including the fringing effects associated with electrode spacing, the minimum resolvable capacitance of a standard laboratory inductance‐capacitance‐resistance (LCR) meter, a suitable distance between the light source and photodiodes, and sufficient fluid volume to ensure development of bulk phase behavior. To determine the optimum balance of these factors, a three‐dimensional electrostatic model of the chamber was constructed in COMSOL Multiphysics 6.1 [38], representing the two stainless‐steel electrodes as parallel plates separated by the fluid and glass layers (see Section S1). The relative error in dielectric constant arising from fringing‐field effects was evaluated across a range of electrode areas and interelectrode gaps (Figure 2a). Smaller gaps and larger areas reduce the relative error toward values below 1%. Based on this trend, a geometry was selected that maintained a measured capacitance above 1 pF, ensuring operation within the high‐accuracy regime of standard laboratory LCR meters, while also maintaining geometric simplicity and practical device assembly. The chamber was designed with an area of 15 cm^2^ and a 0.5 cm gap (total volume equivalent to 7.5 mL). While smaller volumes are in principle achievable with the same measurement approach, doing so would require more sensitive and costly instrumentation to maintain measurement accuracy at reduced capacitance and flow‐rate scales. Furthermore, an optical path length of 2 cm was chosen to balance sensitivity at both low and high turbidity [39], resulting in a chamber width of 2 cm and a height of 7.5 cm. The chamber was constructed from glass to ensure chemical compatibility with a wide range of fluids while allowing unobstructed optical transmission between the light source and detectors.

### 2.2 Capacitance Measurements

In an ideal case, the sensing chamber can be approximated as a series capacitor composed of three dielectric layers, including two glass panes and a central fluid layer. However, the measured capacitance also includes additional contributions, namely parasitic capacitance (arising from air gaps), adhesive layers, wiring, and other components. The equivalent capacitance is given by:

(1) $$C_{\mathrm{measured}}={\left(\frac{2d_{g}}{\varepsilon_{0}\varepsilon_{g}A}+\frac{d}{\varepsilon_{0}\varepsilon_{f}A}\right)}^{-1}+C_{p}$$

where *d_g_* and ε_*g*_ are the thickness and dielectric constant of the glass, *d* and ε_*f*_ are the thickness and dielectric constant of the fluid layer, *A* is the electrode area, ε_0_ is the vacuum permittivity, and *C_p_* is the lumped parasitic capacitance. To determine ε_*g*_ and *C_p_*, calibration measurements were performed using reference fluids, air, and deionized (DI) water. Capacitance measurements were carried out using an LCR meter operated at 100 kHz and 1 V to minimize electrode polarization [40]. For validation, dielectric constants were measured for reference liquids spanning a wide range of chemical classes, including alkanes, esters, alcohols, glycols, and DI water, showing close agreement with literature values within reported ranges (Figure 2b). These measurements were performed on homogeneous fluids, for which the dielectric response is spatially uniform. However, detecting phase separation in mixtures requires leveraging spatial and temporal capacitance changes within a defined sensing region.

By design, the electrodes overlap only a portion of the chamber volume (upper 7 mL), defining a volumetrically asymmetric sensing region where the measurement is sensitive to the presence of phase boundaries, while contributions from fluid outside this region are negligible (Figure 2c). For miscible mixtures, the dielectric response reflects a single, uniform phase within the overlap region, and the capacitance remains constant over time. In contrast, immiscible systems initially contain a mixture of both phases in the sensing region immediately after mixing, and as phase separation proceeds and one phase leaves the overlap region, the effective capacitance changes over time. This time‐dependent deviation of capacitance from its initial post‐mixing value provides the signal used to identify immiscibility, even if the final fluid‐fluid interface ultimately resides outside the overlap region. Because the response depends only on the presence or absence of a phase boundary moving through the sensing region, this method is insensitive to the specific fluid being tested and enables miscibility assessment without requiring prior knowledge of fluid properties or pure‐component reference measurements. For this method, electrodes spanning the full chamber volume would be ineffective, as they would average local phase separation over the entire fluid volume and could fail to detect immiscibility altogether.

### 2.3 Turbidity Measurements

Turbidity was monitored, in accordance with the ISO 7027 standard [41], using an 860 nm light‐emitting source aligned with two silicon photodiodes placed at 90° (nephelometric scatter) and 180° (transmitted light) relative to the beam (detailed specifications in Section S2.1). The wavelength was chosen to coincide with the peak spectral responsivity of the photodiodes and to minimize absorption by typical organic liquids, allowing changes in signal to be attributed primarily to scattering. The light source and the 180° photodiode were positioned 1 cm from the chamber's base in the vertical direction and separated by 2 cm. The 90° photodiode was placed on the bottom of the chamber equidistant from the other diode and the light source. The chamber setup is shown schematically in Figure 2d. Moreover, the chamber was wrapped in black tape to suppress ambient light and internal reflections. This setup provides complementary sensitivity, where the 90° detector is most responsive at low turbidity, whereas the transmitted signal at 180° remains informative at high turbidity [42]. To restrict the light source's illumination angle and reduce direct exposure of the 90° detector, the light output was passed through a 1 mm aperture collimator (Figure 2e). Collimation design and calculations are presented in Section S2.2.

The light source was driven by a constant‑current supply, and photodiode currents were read directly using a multichannel data‑acquisition unit, which provided stable measurements at microampere levels. The input‐output response of both detectors, with an air‐filled chamber, was verified to be linear up to 0.5 A of source current (Figure 2f). An operating current of 0.4 A was selected as a compromise between signal level, linearity, and thermal stability of the emitter. Under clear conditions, the collimated beam produced a negligible signal at the 90° detector (Figure 2f), confirming that the scattered‑light photodiode was not reading direct illumination. Turbidity calibration was performed using aqueous standards of Formazin with known turbidity values spanning 0–4,000 Formazin Nephelometric Units (FNU). For each standard, the 90° and 180° currents were recorded (Figure 2g), and the trends were compared to those reported by Hach Company, shown in Figure S5 [43]. At higher turbidities, multiple‐scattering effects cause the 90° signal to lose monotonicity and the 180° signal to become less sensitive, limiting the usable dynamic range of either channel alone. Using the ratio of 90°/180° readings (Figure 2h) provides an approximately linear response with improved sensitivity across the full turbidity range, reducing sensitivity to multiple‐scattering effects, and follows ISO 7027 recommendations for extending reliable measurements to higher turbidities [41, 44]. In accordance with this standard, turbidity values derived from the 90°/180° ratio are reported in Formazin Nephelometric Ratio Units (FNRU), where 1 FNRU is equivalent to 1 FNU. This ratio is used for quantitative turbidity estimation for subsequent miscibility measurements.

### 2.4 Automation and Full Setup

The chamber was integrated into an automated platform that combines fluid handling, electronic instrumentation, and centralized computer control (Figure S6). Miscibility experiments were executed under computer control using custom Python codes that coordinated the Hamilton syringe pumps, LCR meter, and data acquisition (DAQ) unit as a single automated platform (hardware details in Section S2.3). Multiple sample pumps (three in this work, but readily extendable to additional components) delivered the individual fluids, allowing binary, ternary, or higher‑order mixtures to be prepared without changing the control logic. For each target composition, the script converted the desired volume fractions into withdrawal volumes for a fixed total of 7.5 mL and assigned composition‑dependent injection flow rates proportional to those fractions so that all pumps completed injection simultaneously. The individual streams were combined in a Koflo static inline mixer at a total flow rate of 40 mL/min before entering the chamber, ensuring initial homogenization of the mixture [45]. After a 2 s settling period, the program triggered synchronous acquisition of capacitance and ratio readings at 0.5 s time intervals.

Once the measurement is completed, the outlet pump withdraws a volume exceeding the chamber capacity, thereby removing the liquid sample and drawing air through the chamber to clear residual fluid before the next experiment. Across all fluid pairs tested in this work, the air purge protocol was sufficient to prevent detectable carryover between successive measurements. For chemically aggressive or highly viscous fluids where air purging alone may be insufficient, additional solvent rinse steps can be incorporated into the cleaning sequence using the same automated pumping infrastructure without modifying the control logic, at the cost of approximately 90 s of additional overhead per measurement cycle. The cleaning protocol and its validation are described in detail in Section S2.4 and Figure S7. For temperature‐dependent experiments, a temperature‐controlled water bath (Heidolph Hei‐PLATE Mix n Heat Core+ with PT1000 temperature sensor, ±0.2°C) was used, with fluids preheated externally prior to injection [46].

### 2.5 Data Analysis

There are three primary operating modes: (1) a preset mode, which supports both high‐throughput screening and phase behavior kinetics using predefined mixture lists and/or fixed experiment durations, (2) a GPC mode for ternary miscibility gap mapping, and (3) an NLP mode to determine tie lines (Figure S8). Across all supported modes of running the device, the raw data consists of the 90°/180° ratio and capacitance readings over time, namely *R*(*t*) and *C*(*t*), respectively, sampled at 0.5 s intervals. For the preset and GPC modes, these signals were converted to a simple miscible or immiscible label using fixed thresholds on the change in capacitance and initial ratio. The initial ratio *R*_0_ was obtained from the first measurement point and compared to a threshold of 0.009, which corresponds to approximately 10 FNRU. This cutoff was chosen to be well above the 4 FNRU limit, which is regarded as visibly cloudy by the World Health Organization [47], providing a clear margin that reduces false positives for systems in which refractive‐index contrast and domain morphology produce scattering behavior different from aqueous Formazin standards. Mixtures with *R*_0_ ≥ 0.009 were immediately labeled immiscible, reflecting visibly cloudy systems at the start of the experiment. For mixtures with *R*_0_ < 0.009, we monitored the absolute change in capacitance |Δ*C*(*t*)| = |*C*(*t*) − *C*_0_| , where *C*_0_ is the initial capacitance, and declared a mixture immiscible if |Δ*C*| ≥  0.10 *pF*, a value exceeding the LCR meter noise indicating a meaningful separation of the phases in the sensing region. The optical ratio was evaluated first to provide rapid classification for clearly cloudy systems, with a deliberately conservative threshold to preserve direct‐readout speed and avoid overfitting to specific refractive‐index contrasts, while capacitance deviation resolves weakly scattering and isorefractive mixtures that fall below this threshold.

For mixtures that do not trigger the immiscibility thresholds, the temporal stability of capacitance was monitored every 30 s, with a negligible slope indicating a homogeneous, miscible system. This slope criterion complements the immiscibility thresholds and serves to avoid unnecessary long acquisition times. In preset high‐throughput runs and during GPC mapping, the run stopped autonomously after reaching a miscible/immiscible decision and the system moved on to the next composition/mixture, if available. In contrast, when preset mode was used for phase separation kinetics or emulsion stability studies, data acquisition continued for a predefined duration even after a miscible or immiscible classification was reached, allowing the full temporal evolution of the capacitance and optical signals to be captured.

For ternary mapping in GPC mode, the resulting set of discrete labels on a composition grid was used to infer the miscibility gap. GPC was selected for this task because it models nonlinear miscibility boundaries from limited data while quantifying prediction uncertainty, allowing active learning to target the most informative compositions near the binodal rather than sampling exhaustively. To find the binodal in ternary systems, compositions were first sampled automatically until both miscible and immiscible labels were observed. Once at least one example of each class was available, the accumulated labels were used to initialize a Gaussian process classifier operating on the first two composition coordinates *f*_1_ and *f*_2_, with the third component given by *f*_3_ =  1 − *f*_1_ − *f*_2_. The classifier was then iteratively refined as new measurements were acquired. At each iteration, the classifier predicted the probability of miscibility *P*(*M*) across the diagram, and subsequent experiments were proposed at compositions where *P*(*M*) is closest to regions of maximum model uncertainty and likely proximity to the miscible/immiscible boundary. This active‐learning loop continued until the predicted miscibility field converged and the miscibility gap was resolved. The complete GPC workflow is described in Section S3.1.

In NLP mode, once the miscibility gap was obtained, tie line compositions were calculated using constrained optimization (detailed implementation in Section S3.2). NLP was used because the spatial selectivity of the sensor provides independent signal constraints on each coexisting phase, and the nonlinear relationships between composition, geometry, and sensor response make optimization the natural framework for solving this inverse problem efficiently and reproducibly for each feed composition. For each experiment, a composition within the two‐phase region, selected either randomly or from a predefined list, was prepared and the overall composition was related to candidate top and bottom phase compositions parameterized along the experimentally determined binodal, with the lever rule enforcing mass balance and interface position. Predicted sensor responses were generated from property models obtained during miscible mixtures calibration. Capacitance was evaluated as the sum of the upper and lower phase capacitances weighted by the electrode overlap and the interface height (parallel capacitors, including upper phase and a proportion of the lower phase in the sensing region), whereas the optical ratio was predicted from the lower phase composition using the calibrated turbidity model. The NLP minimizes the mismatch between measured and predicted capacitance, ratio, and interface position subject to constraints on phase fractions, mass balance, and feasible compositions. By restricting solutions to the inferred binodal and accounting for device geometry, this approach yields tie lines consistent with both the miscibility map and the multimodal measurements. The throughput of this approach is limited by the mixture settling time, since measurement and classification are effectively instantaneous once the capacitance and ratio signals stabilize.

## 3 Results and Discussion

### 3.1 Binary Miscibility Benchmarking across Multiple Chemical Classes

**FIGURE 3:**
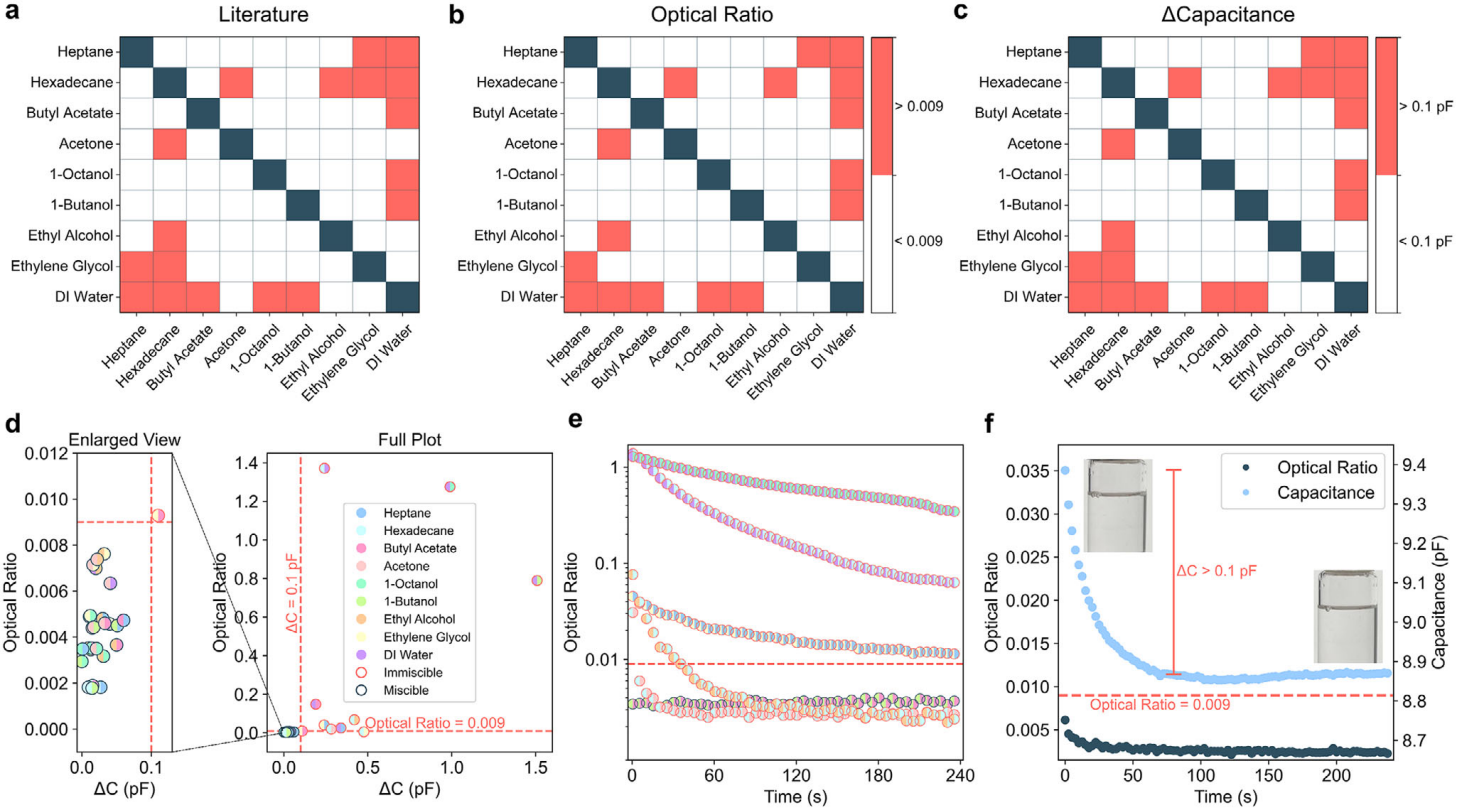
Binary miscibility classification and kinetic characterization. (a) Literature‐based miscibility matrix for nine reference solvents, where red indicates immiscible mixtures. (b) Corresponding experimental classification matrix based on optical ratio threshold. (c) Experimental classification matrix based on capacitance deviation (Δ*C*) threshold. (d) Scatter plot of optical ratio versus Δ*C* for all binary mixtures, with an enlarged view highlighting the cluster of miscible data points below the classification thresholds. Each data point represents one binary pair as a bicolor half‐circle marker, with each half colored according to one of the nine reference solvents in the legend. The border color indicates the miscibility classification. (e) Time evolution of the optical ratio over 4 min for representative mixtures, with fluid pairs colored according to the legend in (d), capturing phase separation kinetics. (f) Transient optical ratio and capacitance responses for the isorefractive ethylene glycol/hexadecane mixture.

The platform's performance was systematically validated across various fluid systems to assess its accuracy in miscibility classification, phase separation, and emulsion stability tracking, partial miscibility behavior, temperature‐dependent miscibility, and determination of binodal curves and tie lines. Binary mixtures at a fixed composition corresponding to a 50:50 volume ratio served as the initial test case for platform validation, with each measurement repeated three times to confirm classification consistency. Nine reference liquids were selected based on their extensive availability in literature data and to span a broad range of chemical classes and property ranges. The set of reference liquids spanned alkanes, esters, ketones, alcohols, glycols, and DI water, with dielectric constants ranging roughly from 2 to 80, and refractive indices ranging from 1.33 to 1.43 (Table S1). The miscibility classification for each of the pairs, assembled from literature data, served as a benchmark (Figure 3a) [48, 49]. Consistent with expectations, DI water and glycols are miscible with short alcohol but immiscible with longer alcohols and alkanes, whereas nonpolar–nonpolar pairs such as heptane/hexadecane are miscible. These trends are directly reflected in our measurements, which use both the optical ratio and the change in capacitance for classification. Although the two metrics are used jointly for classification (exceeding either threshold is sufficient to label a mixture as immiscible), they are presented separately to show the sensitivity of each proxy (Figure 3b,c).

The optical ratio criterion performed well when there was a refractive index contrast between the immiscible phases, while the change in capacitance criterion was accurate for all binary mixtures tested. More specifically, the optical ratio criterion incorrectly labeled the isorefractive immiscible mixture of hexadecane and ethylene glycol as miscible, illustrating the risk of relying solely on turbidimetry for miscibility screening. Reducing the optical threshold to capture this isorefractive system would misclassify three miscible pairs as immiscible, as their refractive index contrast produces scattering signals that fall between the two threshold levels. This trade‐off illustrates the challenge of generalizing a single optical threshold across chemically diverse systems: a threshold conservative enough to avoid false positives in high‐contrast miscible pairs will inevitably miss low contrast immiscible ones, while a more sensitive threshold introduces false positives. Capacitance deviation resolves this without threshold compromise, as it responds to the physical redistribution of phases rather than their optical contrast. Furthermore, these results demonstrate that absolute dielectric constant values alone are an unreliable proxy for the like‐dissolves‐like principle. Several fully miscible liquid pairs in this study exhibit large differences in dielectric constant, while some immiscible combinations differ only modestly. Hence, using capacitance deviation upon mixing provides a more accurate classification, as further demonstrated across additional isorefractive binary combinations in Figure S9.

The change in capacitance over 4 min and the initial optical ratio were recorded for all tested mixtures (Figure 3d). This 4‐min timeframe captures both miscibility classification and phase separation dynamics, relevant to the next section. Miscible systems cluster near the origin, indicating low optical scattering, high transmission, and stable dielectric constant, while immiscible mixtures are scattered in regions exceeding thresholds (shown in red dashed lines), reflecting either immediate cloudiness or phase separation. Notably, several immiscible systems, including butyl acetate/ethylene glycol and DI water/heptane, fall within close proximity to the optical threshold yet are unambiguously identified as immiscible by capacitance. The conservative optical threshold is intentionally set to minimize false positives from miscible systems with high refractive index contrast, while still capturing the majority of immiscible systems through direct evaluation. Capacitance then identifies the remaining immiscible systems that fall below this conservative threshold, ensuring complete classification. Rather than serving identical roles, the dual modality approach operates in a complementary manner, enabling rapid and robust classification across chemically diverse systems. The platform was further validated on additional fluid classes, including highly viscous fluids and salty systems, with results presented in Figure S10.

### 3.2 Phase Separation Kinetics and Emulsion Persistence

Beyond the miscible/immiscible classification, all mixtures were evaluated for 4 min to examine phase separation kinetics. The 4‐min measurement window is sufficient to capture and differentiate the kinetic behaviors exhibited by different fluid pairs, from rapid phase separation to sustained emulsion formation, though longer studies, such as the 12‐h measurement presented in Figure S11, can be conducted when needed. These kinetic behaviors are distinguished by their temporal signal characteristics. Phase separation describes the evolution of an initially dispersed mixture toward distinct bulk phases, characterized by steep temporal capacitance and optical ratio signals reflecting droplet coalescence and gravitational settling. In contrast, emulsion stability reflects the persistence of a dispersed state, characterized by sustained cloudiness with a stable above‐threshold optical ratio signal.

These distinct behaviors are illustrated through the time‐dependent optical ratio responses of six different mixtures (Figure 3e). These mixtures span two orders of magnitude in optical ratio evolution under identical mixing conditions. Water‐based systems illustrate this variability, with water/hexadecane and water/1‐octanol mixtures both exhibiting high initial cloudiness, but the former displaying relatively faster phase separation. Similarly, hexadecane‐based mixtures further validate the platform's capacity to differentiate separation kinetics across diverse systems. Pairing hexadecane with acetone, ethyl alcohol, and water yields progressively slower phase separation, with acetone reaching complete separation within 40 s and ethyl alcohol by 90 s, both evident by slope stabilization, while water results in persistent emulsification throughout the measurement window. Optical ratio signals of water/hexadecane, water/1‐octanol, and ethylene glycol/heptane mixtures remain above the threshold throughout the measurement, indicative of visible cloudiness persisting beyond 4 min. However, the ethylene glycol/heptane exhibited slight stabilization above the threshold, suggesting near‐complete separation with a turbid bottom phase. The miscible 1‐butanol/butyl acetate mixture maintains a stable low optical ratio throughout, characteristic of a homogeneous single‐phase system. These measurements confirm that the platform distinguishes between rapid settling and more persistent, stabilized dispersions in immiscible systems. The time‐resolved profiles provide a basis for application‐driven assessment, where quantitative metrics such as settling time or signal slope can be defined against application‐specific thresholds to guide selection or rejection of fluid combinations in downstream workflows. Photographs of all six representative binary mixtures initially and after 4 min are provided in Figure S12 for qualitative reference.

Capacitance and optical ratio values were recorded as a function of time for the isorefractive hexadecane/ethylene glycol mixture (Figure 3f). Both signals displayed a similar trend, with an initial steep decrease followed by stabilization after approximately 1 min. The optical ratio remained below the ratio threshold throughout the measurement, reflecting the minimal difference in refractive index between the two fluids, and consequently passed the ratio‐based miscibility test. In contrast, the capacitance signal revealed a decrease that noticeably exceeded the |Δ*C*| =  0.10 pF threshold. This behavior indicates phase separation in which the ethylene glycol‐rich phase settles toward the bottom of the chamber, reducing the effective dielectric constant within the effective‐electrode‐sensing region and leading to a measurable drop in capacitance over time. Inset photographs (Figure 3f) show the hexadecane/ethylene glycol mixture initially and after 4 min, illustrating that this system is challenging to distinguish visually both before and after phase separation, despite its clear failure in the capacitance‐based threshold.

### 3.3 Partial Miscibility and Temperature‐Dependent Phase Boundaries

**FIGURE 4:**
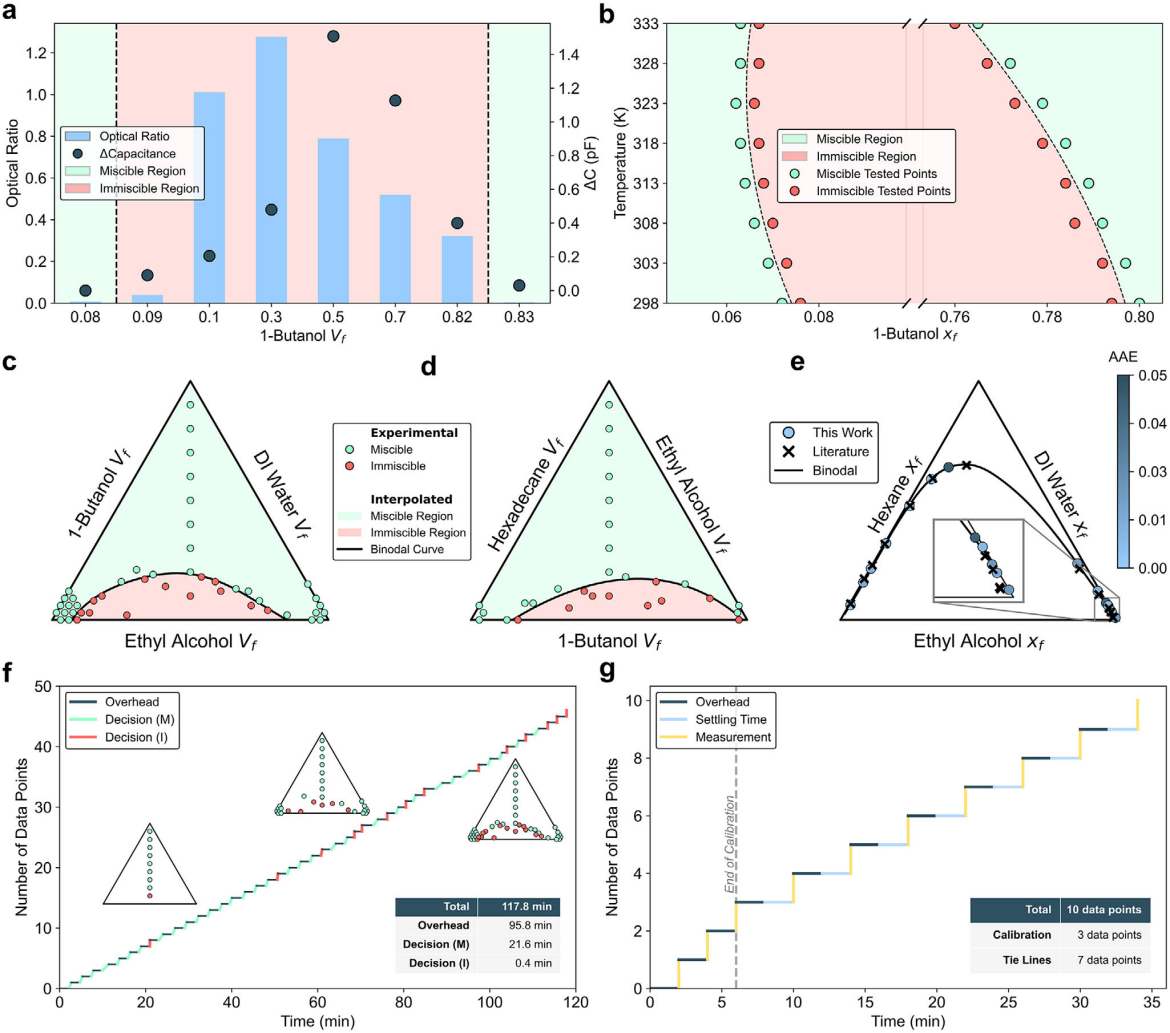
Ternary phase equilibrium characterization and throughput analysis. (a) Optical ratio and Δ*C* response for the 1‐butanol/water system across compositions. (b) Temperature‐dependent phase diagram for 1‐butanol/water mixture. Ternary map for the (c) ethyl alcohol/1‐butanol/water and (d) ethyl alcohol/1‐butanol/ hexadecane systems, showing experimentally determined binodal curves. (e) Tie line mapping results compared with literature, with deviation quantified by the average absolute error. (f) Time‐resolved binodal acquisition showing cumulative mapped points and time allocation, where overhead corresponds to pumping and withdrawal steps. (g) Tie line mapping throughput as cumulative tie line data points versus experiment time.

The final binary case examined was the partially miscible mixture of 1‐butanol and DI water, where partial miscibility is defined by systems that form a single phase at certain compositions but split into two phases at others. We tested a series of volume fractions to locate the compositions at which the classification flips between miscible and immiscible (Figure 4a), with the boundaries found in close agreement with literature data [15]. At compositions outside the miscibility window (1‐butanol volume fraction: *v_f_* < 0.09 or *v_f_* > 0.82), both the ratio and |Δ*C*| remained near zero, consistent with homogenous phase behavior. Within the two‐phase region, both properties increased to levels well beyond their respective thresholds. The rise in optical ratio is due to light scattering from the dispersed phase, along with lower light transmission, while capacitance experiences larger drops as the composition of the phases within the electrode overlap region changes. The bell‐shaped dependence of both signals on composition, with maxima toward the middle of the immiscible window and minima near the binodal boundaries, is characteristic of partially miscible systems and demonstrates that both signals can localize the onset of phase separation on either side of the miscibility gap. Specifically, closer to the binodal, there is less of the dispersed phase, and hence the scattering is reduced. Similarly, the larger change in capacitance observed in the middle of the immiscible region correlates with a greater amount of the denser liquid, water, separating and settling toward the bottom of the chamber, while closer to the binodal, there is a smaller extent of phase separation.

The same approach was then used to map the temperature‐dependent phase diagram of the water/1‐butanol system (Figure 4b). For this phase diagram, compositions are stated in molar fractions due to the temperature dependency of volume fractions. The sensing device was placed on a temperature‐controlled water bath, while the fluids were heated externally prior to injection. Experiments were performed in temperature increments, with several compositions tested at each temperature. At each temperature, the transition between miscible and immiscible behavior was identified using the same thresholds, and the corresponding binodal points were estimated by fitting a boundary between the compositions exhibiting a classification flip. The measured phase diagram closely aligns with the literature binodal on both the water‐rich and butanol‐rich sides, capturing the narrowing of the two‐phase region as the temperature approaches the critical point [15].

### 3.4 Active‐Learning Binodal Mapping and NLP‐Based Tie Line Extraction

Ternary systems were tested using the platform complemented with GPC‐based active learning to determine the binodal curve. For the ethyl alcohol/1‐butanol/water system (Figure 4c), the autonomous mapping reproduced the literature binodal curve with close agreement [50]. We computed the absolute average error in volume fraction between our measured compositions and the 15 equilibrium compositions reported in the cited literature, yielding an average composition error of 0.01 (calculation method in Section S3.3). Six of those 15 compositions fell effectively on the binodal with zero deviation in a 0.01 composition grid spacing, and the maximum deviation observed was 0.03. The literature compositions and the calculation of composition errors are reported in Table S2. The composition‐error metric can be applied in reverse. After the binodal has been mapped, the model accepts any target composition and calculates which component or components and the required amounts to convert that composition to a one‐phase or two‐phase state. This reverse‐mapping capability supports formulation work and experiment planning, with the mathematical framework outlined in Section S3.4. The ethyl alcohol/1‐butanol/hexadecane system (Figure 4d) represents a previously unmapped ternary phase diagram, with the platform‐determined binodal validated through visual inspection of phase separation at selected compositions. Both ternary phase diagrams were mapped without prior knowledge of the system, the individual components, or any calibration.

Beyond binodal curve mapping, the platform enables tie line determination through NLP, providing complete thermodynamic characterization of ternary liquid‐liquid equilibria. For the ethyl alcohol/hexane/water system, three pure‐component calibration measurements were used to construct sensor prediction models relating capacitance and turbidity to composition. Seven feed compositions within the immiscible region were then sequentially tested by the system, with each mixture allowed to settle to equilibrium before measurement to determine the top and bottom phase compositions. Comparison of these platform‐determined tie lines against literature values for the same feed compositions (Figure 4e) reveals an average absolute error of 0.01 in molar fraction across all seven tie lines [51]. The error distribution shows that most phase compositions are determined within 0.02, with one tie line exhibiting a relatively higher error of 0.05. This elevated error corresponds to the tie line located deepest within the ternary composition space, which is the furthest from all three pure‐component calibration points. This region is therefore least accurately described by the edge‐based sensor models, resulting in the larger deviation observed. The framework used three calibration points and seven feed compositions in this demonstration, though both can be adjusted based on application needs, with three calibration points representing the minimum requirement. Additional calibration mixtures beyond the three pure components could improve accuracy in undersampled compositional regions but would increase total measurement time per tie line. For applications requiring tighter accuracy across all compositional regions, inclusion of one or two interior ternary calibration points would directly address this limitation, representing a trade‐off between accuracy and throughput that can be adjusted based on application requirements. The framework also utilizes component density and molecular weight data to convert volume fractions to molar fractions for lever rule calculations. Volume fractions alone could be used, which would assume ideal mixing behavior, while the density‐based conversion enables accurate characterization of non‐ideal systems.

The throughput of the autonomous mapping workflow was quantified for the ethyl alcohol/1‐butanol/water system (Figure 4f). Complete ternary phase diagram construction via GPC required approximately 40 measurement points and approximately 2 h total time, representing a 14‐fold reduction compared to exhaustive grid‐based sampling, which would require 595 measurements for an equivalent resolution of 0.03. At an average measurement time of approximately 3 min per composition, exhaustive grid sampling would have required approximately 30 h, rendering it impractical even for a high‐throughput platform and making active learning essential for efficient ternary mapping. In this approach, the active learning strategy converges adaptively based on model uncertainty rather than a predetermined grid, meaning the number of required measurements varies by system complexity. The scan line method tests along a diagonal composition path, refining sampling density when encountering immiscibility or contrasting classification labels along the traverse. Miscible classifications require extended observation to confirm signal stability rather than immediate visual assessment, with the majority of miscible points achieving stable signals within 30 s. Immiscible compositions enable relatively faster screening than miscible systems, as they trigger immediate classification upon cloudiness or capacitance deviation. Of the 14 immiscible points identified in this system, two were detected by capacitance deviation and 12 by the optical ratio threshold. The two capacitance‐detected points correspond to low‐cloudiness mixtures that fell below the turbidity threshold before the 2 s wait period allowed prior to measurement, demonstrating the complementary nature of dual‐modality sensing in capturing phase separation across diverse optical characteristics. The ethyl alcohol/1‐butanol/hexadecane system required 29 measurements and 71 min in total (Figure S13).

Throughput for tie line determination was evaluated using the ethyl alcohol/hexane/water system (Figure 4g). Calibration of the three pure components required 6 min total, with each additional calibration point requiring 2 additional minutes. Calibration measurements are instantaneous as they involve homogeneous compositions requiring no settling time, with the 2‐min duration per point attributed solely to overhead for pumping, sample withdrawal, and cleaning, which is common to both binodal mapping and tie line workflows. Immiscible mixture settling time, defined by the time it takes a heterogeneous mixture to separate into two phases, represents a large and variable component of the measurement cycle, with the ethyl alcohol‐hexane‐water system reaching equilibrium within roughly 2 min per feed composition. Systems exhibiting persistent cloudiness and slower phase separation would require correspondingly longer measurement times, as throughput scales directly with system‐specific separation kinetics. Overhead for pumping, sample withdrawal, and cleaning averaged approximately 2 min per measurement. Once equilibrium is reached, tie line compositions are determined instantaneously from dual‐modality measurements via the NLP framework. The complete workflow, including three pure‐component calibrations and seven feed compositions, required 34 min total, yielding approximately 5 min per tie line.

## 4 Conclusion

We developed an automated, high‐throughput, and continuous capacitance‐turbidity platform that enables (im)miscibility characterization. The platform overcomes limitations of traditional single‐modality proxies through complementary dual and asymmetric sensing. Turbidity measurements with a conservative threshold enabled rapid direct evaluation for the majority of systems. Post‐mixing capacitance deviations, independent of pure‐component data, mixing rules, and the chemical generality constraints of absolute dielectric values, ensured accurate classification of isorefractive mixtures and systems falling near the optical threshold. Unlike approaches that rely on absolute dielectric constant as a miscibility proxy, the deviation‐based signal responds directly to the physical redistribution of phases, making it inherently generalizable across chemical systems without prior property knowledge. Beyond miscibility labeling, time‐resolved measurements unlock characterization of phase behavior, resolving phase separation kinetics and distinguishing rapid settling from stabilized emulsions. Coupling this dual‐modality framework with Gaussian‐process‐based active learning enables autonomous mapping of ternary phase diagrams in approximately 2 h, while NLP extracts tie lines in about 5 min per line. This timeframe is approximately four times faster than Raman‐based methods, which additionally require prior binodal knowledge from a separate experiment; here, the binodal is instead determined autonomously by the same platform within the same workflow.

This characterization capability directly addresses the scalability requirements of self‐driving labs and autonomous discovery platforms, which demand rapid phase behavior data at the pace at which new formulation candidates are generated. Together, miscibility labels, kinetics, and ternary mapping create data streams for training predictive models and constraining formulation or separation design spaces. In both formulation and separation workflows, these measurements serve distinct but complementary roles: eliminating inadmissible compositions before downstream property optimization or providing binodal and tie line data as direct target outputs. Such capability is particularly relevant for emerging areas such as sustainable solvent systems and next‐generation functional fluids, where existing phase equilibrium data are often sparse or unavailable. Future extensions to elevated pressures and broader temperature ranges would bring supercritical and liquefied‐gas systems within reach, and integration with optimization algorithms targeting application‐specific performance metrics would further expand the platform's utility. Ultimately, by bridging the gap between high‐throughput formulation and physical characterization across diverse chemical systems, this work establishes a pathway toward the fully autonomous design of complex liquid systems.

## Author Contributions

**Tarek Eid**: writing – original draft, writing – review and editing, visualization, validation, methodology, investigation, formal analysis, data curation, conceptualization. **Maryam Ebrahimiazar**: writing – review and editing, writing – original draft, visualization, methodology, formal analysis, conceptualization. **Mohammad Zargartalebi**: writing – review and editing, writing – original draft, visualization, supervision, resources, project administration, methodology, funding acquisition, conceptualization. **David Sinton**: writing – review and editing, supervision, resources, funding acquisition.

## Conflicts of Interest

The authors declare no conflicts of interest.

## Supporting information




**Supporting File**: advs76294‐sup‐0001‐SuppMat.pdf.

## Data Availability

The data that support the findings of this study are available from the corresponding author upon reasonable request.

## References

[1] E. Conte , R. Gani , and K. M. Ng , “Design of Formulated Products: A Systematic Methodology,” AIChE Journal 57, no. 9 (2011): 2431–2449, 10.1002/aic.12458.

[2] G. M. Kontogeorgis , S. Jhamb , X. Liang , and K. Dam‐Johansen , “Computer‐aided Design of Formulated Products,” Current Opinion in Colloid & Interface Science 57 (2022): 101536, 10.1016/j.cocis.2021.101536.

[3] Q. Ma , Y. Song , W. Sun , et al., “Cell‐Inspired all‐Aqueous Microfluidics: From Intracellular Liquid–Liquid Phase Separation toward Advanced Biomaterials,” Advanced Science 7, no. 7 (2020): 1903359, 10.1002/advs.201903359. PMC7141073 32274317

[4] H. Tao , C. Rigoni , H. Li , et al., “Thermodynamically Controlled Multiphase Separation of Heterogeneous Liquid Crystal Colloids,” Nature Communications 14, no. 1 (2023): 5277, 10.1038/s41467-023-41054-7. PMC10465492 37644027

[5] S. Jiang , J. Zhang , K. Diao , X. Liu , and Z. Ding , “Research Advances in Solvent Extraction of Lithium: The Potential of Ionic Liquids,” Advanced Functional Materials 35, no. 29 (2025): 2423566, 10.1002/adfm.202423566.

[6] Q. Yu , Y. Wang , G. Huang , et al., “Task‐Specific Oil‐Miscible Ionic Liquids Lubricate Steel/Light Metal Alloy: A Tribochemistry Study,” Advanced Materials Interfaces 5, no. 19 (2018): 1800791, 10.1002/admi.201800791.

[7] W. Wang , H. Wang , Z. Zhang , et al., “In Situ Liquid‐Liquid Phase Separation of Peptides into Droplets Targeting Membraneless Organelles for Enhanced Cancer Chemotherapy,” Advanced Materials 37, no. 28 (2025): 2420399, 10.1002/adma.202420399. 40331504

[8] P. Navalpotro , C. M. S. S. Neves , J. Palma , M. G. Freire , J. A. P. Coutinho , and R. Marcilla , “Pioneering Use of Ionic Liquid‐Based Aqueous Biphasic Systems as Membrane‐Free Batteries,” Advanced Science 5, no. 10 (2018): 1800576, 10.1002/advs.201800576. PMC6193149 30356931

[9] F. Ravera , K. Dziza , E. Santini , L. Cristofolini , and L. Liggieri , “Emulsification and Emulsion Stability: The Role of the Interfacial Properties,” Advances in Colloid and Interface Science 288 (2021): 102344, 10.1016/j.cis.2020.102344. 33359938

[10] J. Wang , X. Yang , J. J. Klemeš , K. Tian , T. Ma , and B. Sunden , “A Review on Nanofluid Stability: Preparation and Application,” Renewable and Sustainable Energy Reviews 188 (2023): 113854, 10.1016/j.rser.2023.113854.

[11] M. Abolhasani and E. Kumacheva , “The Rise of Self‐driving Labs in Chemical and Materials Sciences,” Nature Synthesis 2, no. 6 (2023): 483–492, 10.1038/s44160-022-00231-0.

[12] J. Li , Y. Tu , R. Liu , Y. Lu , and X. Zhu , “Toward “on‐Demand” Materials Synthesis and Scientific Discovery through Intelligent Robots,” Advanced Science 7, no. 7 (2020): 1901957, 10.1002/advs.201901957. PMC7141037 32274293

[13] N. Mukhin , P. Jha , and M. Abolhasani , “The Role of Flow Chemistry in Self‐Driving Labs,” Matter 8, no. 7 (2025): 102205, 10.1016/j.matt.2025.102205.

[14] A. Skrzecz , D. G. Shaw , A. Maczynski , and A. Skrzecz , “IUPAC‐NIST Solubility Data Series 69. Ternary Alcohol–Hydrocarbon–Water Systems,” Journal of Physical and Chemical Reference Data 28, no. 4 (1999): 983–1235, 10.1063/1.556052.

[15] A. Maczynski , D. G. Shaw , M. Goral , and B. Wisniewska‐Goclowska , “IUPAC‐NIST Solubility Data Series. 82. Alcohols With Water—Revised and Updated: Part 1. C4 Alcohols With Water,” Journal of Physical and Chemical Reference Data 36, no. 1 (2007): 59–132, 10.1063/1.2366707.

[16] A. Mączyński , D. G. Shaw , M. Góral , and B. Wiśniewska‐Gocłowska , “IUPAC‐NIST Solubility Data Series. 86. Ethers and Ketones With Water. Part 1. C2–C5 Ethers With Water,” Journal of Physical and Chemical Reference Data 37, no. 2 (2008): 1119–1146, 10.1063/1.2838022.

[17] M. Góral , D. G. Shaw , A. Mączyński , B. Wiśniewska‐Gocłowska , and A. Jezierski , “IUPAC‐NIST Solubility Data Series. 88. Esters With Water—Revised and Updated. Part 1. C2 to C4 Esters,” Journal of Physical and Chemical Reference Data 38, no. 4 (2009): 1093–1127, 10.1063/1.3243853.

[18] C. F. Poole , “Milestones in the Development of Liquid‐Phase Extraction Techniques,” In Liquid‐Phase Extraction, (Elsevier, 2020), 1–44.

[19] J. Gregory , “Turbidity and Beyond,” Filtration & Separation 35, no. 1 (1998): 63–67, 10.1016/S0015-1882(97)83117-5.

[20] S. Krause , “Polymer‐Polymer Miscibility,” Pure and Applied Chemistry 58, no. 12 (1986): 1553–1560, 10.1351/pac198658121553.

[21] C. Linke and S. Drusch , “Turbidity in Oil‐in‐water‐emulsions — Key Factors and Visual Perception,” Food Research International 89 (2016): 202–210, 10.1016/j.foodres.2016.07.019. 28460906

[22] S. Duffield , L. Da Vià , A. C. Bellman , and F. Chiti , “Automated High‐Throughput Partition Coefficient Determination With Image Analysis for Rapid Reaction Workup Process Development and Modeling,” Organic Process Research & Development 25, no. 12 (2021): 2738–2746, 10.1021/acs.oprd.1c00343.

[23] J. J. Wiedmann , Y. N. Demirdögen , S. Schmidt , et al., “Nanoliter Scale Parallel Liquid–Liquid Extraction for High‐Throughput Purification on a Droplet Microarray,” Small 19, no. 9 (2023): 2204512, 10.1002/smll.202204512. 36538723

[24] R. El‐khawaldeh , A. Mandal , N. Yoshikawa , et al., “From Eyes to Cameras: Computer Vision for High‐Throughput Liquid‐liquid Separation,” Device 2, no. 7 (2024): 100404, 10.1016/j.device.2024.100404.

[25] L. J. Augustine , Y. Wang , S. L. Adelman , et al., “Advancing Rare‐Earth (4 f) and Actinide (5 Advancing Rare‐Earth (4 f ) and Actinide (5 f ) Separation through Machine Learning and Automated High‐Throughput Experiments,” ACS Sustainable Chemistry & Engineering 12, no. 45 (2024): 16692–16699, 10.1021/acssuschemeng.4c06166. PMC11558677 39545104

[26] W. Feng , E. Ueda , and P. A. Levkin , “Droplet Microarrays: From Surface Patterning to High‐Throughput Applications,” Advanced Materials 30, no. 20 (2018): 1706111, 10.1002/adma.201706111. 29572971

[27] K. Duch , M. Illner , and J. U. Repke , “Development of an Automated High‐Throughput Screening Platform for the Dynamic Phase Separation Analysis of Microemulsion Systems With AI Image Recognition,” Organic Process Research & Development 29, no. 6 (2025): 1495–1508, 10.1021/acs.oprd.5c00083.

[28] J. Thien , L. Reinpold , T. Brands , H. J. Koß , and A. Bardow , “Automated Physical Property Measurements from Calibration to Data Analysis: Microfluidic Platform for Liquid–Liquid Equilibrium Using Raman Microspectroscopy,” Journal of Chemical & Engineering Data 65, no. 2 (2020): 319–327, 10.1021/acs.jced.9b00636.

[29] M. Kasterke , J. Thien , C. Flake , et al., “Automated Measurement of Liquid‐liquid Equilibria Using Raman Spectroscopy and Single Droplet Tracking in Microfluidic Plug Flow,” Fluid Phase Equilibria 567 (2023): 113718, 10.1016/j.fluid.2022.113718.

[30] M. Hübner and M. Minceva , “Microfluidics Approach for Determination of the Equilibrium Phase Composition in Multicomponent Biphasic Liquid Systems,” Chemical Engineering Research and Design 184 (2022): 592–602, 10.1016/j.cherd.2022.06.023.

[31] B. Zhuang , G. Ramanauskaite , Z. Y. Koa , and Z. G. Wang , “Like Dissolves Like: A First‐principles Theory for Predicting Liquid Miscibility and Mixture Dielectric Constant,” Science Advances 7, no. 7 (2021): abe7275, 10.1126/sciadv.abe7275. PMC7880597 33579702

[32] H. Wang and W. Hou , “Correlations of Surface Free Energy and Solubility Parameters With Dielectric Constant, Refractive Index, and Density for Liquids,” The Journal of Physical Chemistry B 128, no. 22 (2024): 5489–5499, 10.1021/acs.jpcb.4c00581. 38777626

[33] K. Wang , D. Peng , A. Alhadid , and M. Minceva , “Assessment of COSMO‐RS for Predicting Liquid–Liquid Equilibrium in Systems Containing Deep Eutectic Solvents,” Industrial & Engineering Chemistry Research 63, no. 25 (2024): 11110–11120, 10.1021/acs.iecr.4c00796.

[34] I. Antolović , S. Stephan , and J. Vrabec , “High‐throughput Application and Evaluation of the COSMO‐SAC Model for Predictions of Liquid–liquid Equilibria,” Digital Discovery 4, no. 11 (2025): 3191–3207, 10.1039/D5DD00259A.

[35] M. Seifrid , R. Pollice , A. Aguilar‐Granda , et al., “Autonomous Chemical Experiments: Challenges and Perspectives on Establishing a Self‐Driving Lab,” Accounts of Chemical Research 55, no. 17 (2022): 2454–2466, 10.1021/acs.accounts.2c00220. PMC9454899 35948428

[36] F. H. Vermeire and W. H. Green , “Transfer Learning for Solvation Free Energies: From Quantum Chemistry to Experiments,” Chemical Engineering Journal 418 (2021): 129307, 10.1016/j.cej.2021.129307.

[37] O. Bayley , E. Savino , A. Slattery , and T. Noël , “Autonomous Chemistry: Navigating Self‐Driving Labs in Chemical and Material Sciences,” Matter 7, no. 7 (2024): 2382–2398, 10.1016/j.matt.2024.06.003.

[38] COMSOL Multiphysics , www.comsol.com (2025).

[39] C. H. Chu , Y. X. Lin , C. K. Liu , and M. C. Lai , “Development of Innovative Online Modularized Device for Turbidity Monitoring,” Sensors 23, no. 6 (2023): 3073, 10.3390/s23063073. PMC10051103 36991784

[40] D. Padmaraj , J. H. Miller , J. Wosik , and W. Zagozdzon‐Wosik , “Reduction of Electrode Polarization Capacitance in Low‐Frequency Impedance Spectroscopy by Using Mesh Electrodes,” Biosensors and Bioelectronics 29, no. 1 (2011): 13–17, 10.1016/j.bios.2011.06.050. 21872464

[41] ISO , Water Quality — Determination of Turbidity, (International Organization for Standardization, 2019).

[42] T. Matos , M. S. Martins , R. Henriques , and L. M. Goncalves , “A Review of Methods and Instruments to Monitor Turbidity and Suspended Sediment Concentration,” Journal of Water Process Engineering 64 (2024): 105624, 10.1016/j.jwpe.2024.105624.

[43] M. J. Sadar , Turbidity Science, Hach Company Technical Information Series, Booklet No. 11, 1998, printed in U.S.A.

[44] C. W. Anderson , “Chapter A6. Section 6.7. Turbidity,” Techniques of Water‐Resources Investigations, (U.S. Geological Survey, 2005), 10.3133/twri09A6.7.

[45] “StratosTM Static Tube Mixer,” Koflo Corporation (n.d.), https://www.koflo.com/static‐mixers/stock‐static‐mixers/stratos‐tube‐static‐mixers/.

[46] “Hei‐PLATE Mix ‘n’ Heat Core+ (135 mm) Technical Datasheet,” Heidolph North America, LLC (n.d.), https://heidolph.com/america/en/hei‐plate‐mix‐n‐heat‐core‐135‐mm~p352.

[47] Water Quality and Health: Review of Turbidity: Information for Regulators and Water Suppliers (World Health Organization, 2017).

[48] P. C. Sadek , “Book Review: The HPLC Solvent Guide,” in Analytical and Bioanalytical Chemistry, 2nd Edition, (Wiley, 2002), 26–27, 10.1007/s00216-003-2261-y.

[49] M. L. Huber , CRC Handbook of Chemistry and Physics, 105th Edition, (CRC Press, 2024).

[50] F. Ruiz , D. Prats , and V. Gomis , “Quaternary Liquid‐Liquid Equilibrium. Water‐ethanol‐1‐butanol‐chloroform at 25.degree.C. Experimental Determination and Graphical Representation of Equilibrium Data,” Journal of Chemical & Engineering Data 29, no. 2 (1984): 147–151, 10.1021/je00036a015.

[51] T. Moriyoshi , Y. Uosaki , H. Matsuura , and W. Nishimoto , “(Liquid + liquid) equilibria of (water + ethanol + n‐hexane) from 0.1 to 200 MPa at 298.15 K,” The Journal of Chemical Thermodynamics 20, no. 5 (1988): 551–557, 10.1016/0021-9614(88)90083-3.

