# Supplementary material for: Autonomous High‐Throughput Characterization of Liquid‐Liquid Phase Behavior
Source: Adv Sci (Weinh). 2026 Jun 30;13(52):e76294. doi: 10.1002/advs.76294 (PMC13336837; doi:10.1002/advs.76294)
Supplement: Supplementary file 1 [file ADVS-13-e76294-s001.pdf]

Supplementary Information

Autonomous High-Throughput Characterization of Liquid-Liquid Phase Behavior

Tarek Eid[a], Maryam Ebrahimiazar[a], Mohammad Zargartalebi[a,*], David Sinton[a,*]

[a] Department of Mechanical and Industrial Engineering, University of Toronto, 5 King's College Rd., Toronto, Ontario, Canada, M5S 3G8

[*] Corresponding authors. Email: mohammad.zargartalebi@utoronto.ca; dave.sinton@utoronto.ca

Supplementary Notes

S1. Fringing Effects in Capacitors

We used COMSOL Multiphysics to model the electrostatic behavior of a parallel plate capacitor and quantify the effect of fringing fields on dielectric constant extraction. While we are primarily interested in capacitance for phase behavior analysis, we use dielectric constant to validate our capacitance readings both experimentally and in simulation, as the two are directly related through the geometry via:

$$C = \frac{\varepsilon_0 \varepsilon_r A}{d} \tag{S1}$$

where ε_0 is absolute permittivity, ε_r is the dielectric constant of the fluid, A is the area of the electrodes and d is the interelectrode gap. The simulation setup follows that of the reference,[1] in which two metal electrodes are enclosed within a spherical air domain, with one electrode held at 1 V and the other at ground (0 V) using the Electrostatics interface with Terminal boundary conditions. To adapt this to our sensing geometry, we introduced a fluid body filling the gap between the electrodes (Fig. S1). A broad parametric sweep over electrode area and gap distance was then performed to map the fringing behavior across a wide range of geometries. The fringing was quantified as the fraction of total internal energy, U, stored outside the fluid domain:

$$Fringing = \frac{U_{air}}{U_{air} + U_{fluid}} \tag{S2}$$

The dielectric constant was extracted from the energy stored in the fluid domain alone using the following equation:

$$C_{fluid} = \frac{2 \times U_{fluid}}{V^2} \tag{S3}$$

followed by Equation S1. The results for a coarse parametric sweep (5 cm^2 increments in electrode area and 0.1 cm increments in interelectrode gap) are summarized across three assigned dielectric constants (Fig. S2), with a finer sweep presented in Section 2.1 of the main text. Moreover, the addition of a glass layer and electrode asymmetry had negligible effect on the error.

S2. Experimental Setup

S2.1 Emitter and Photodiodes

The infrared emitter and photodetector were both purchased from Digi-Key. The emitter selected was the ams-OSRAM SFH-4718AS-A01,[2] an infrared LED with a peak emission wavelength of 860 nm (Fig. S3) and a typical radiant intensity of 1,350 mW/sr at a forward current of 1 A. Compared to similar emitters with equivalent intensities, the SFH-4718AS-A01 offers a relatively narrow half-angle viewing angle of 25°, which reduces beam divergence and is favorable for directional transmission through the sample. The photodetector was the ams-OSRAM BPW-34 S-Z,[3] a silicon PIN photodiode with a broad spectral sensitivity range of 420–1,120 nm and a peak sensitivity at approximately 900 nm (Fig. S4), well-matched to the emitter. The photodetector features a large radiant-sensitive area of 7.02 mm^2, and a responsivity of 0.62 A/W at 850 nm, confirming strong sensitivity at the emitter's peak wavelength. Additionally, both parameters determine the detected photocurrent using the following equation:

$$I_{pd} = \frac{I_e A_{pd} R}{d^2} \quad \text{(S4)}$$

where larger area, A_{pd}, and higher responsivity, R, yield a stronger signal, I_{pd}, when distance, d, and input current, I_e, are constant, facilitating detection. However, the photodiode's half-angle viewing angle of 60° is relatively wide, meaning it can accept light from off-axis directions, which risks detecting transmitted rather than scattered light for nephelometric measurements. To mitigate this, a collimation system was implemented.

S2.2 Beam Collimation

A 1.6-mm-thick copper block with a 1 mm aperture hole was placed directly adjacent to the emitter to collimate the beam. The block thickness constrains the effective exit half-angle:

$$\theta_{eff} = arctan\left(\frac{r_{ap}}{t}\right) \quad \text{(S5)}$$

where $r_{ap} = 0.5$ mm is the aperture radius and $t = 1.6$ mm is the block thickness, giving $\theta_{eff} \approx 17.4°$. The beam radius at the 90° detector position is then:

$$r_{beam} = r_{ap} + L\, tan\,(\theta_{eff}) \quad \text{(S6)}$$

where $L =$ 14.1 mm is the direct distance from the aperture to the 90° detector. This gives r_{beam} = 4.9 mm, well within the 9.5 mm separation between the beam axis and the 90° photodiode, ensuring that directly transmitted light cannot geometrically reach it.

S2.3 Data Acquisition and Measurement Hardware

Photodiode current readings were acquired using the Keysight 34970A Data Acquisition/Switch Unit equipped with the 34901A multiplexer module,[4] selected for its ability to measure current directly, eliminating the need for an external transimpedance amplifier and simplifying the circuit. Capacitance measurements were performed using the Matrix MCR-6100A LCR meter,[5] selected for its wide frequency range up to 100 kHz, enabling measurements at frequencies that minimize electrode polarization effects, with a measurement accuracy of approximately 0.4% for the impedance range relevant to this study.

S2.4 Chamber Cleaning Protocol

Between successive measurements, the chamber was cleared by withdrawing the fluid sample and drawing air through the chamber using the outlet pump. For the majority of fluid systems tested, particularly during ternary phase diagram mapping, where the same set of components is repeatedly cycled through the chamber, air purging alone produced no detectable carryover between measurements. The vertical chamber geometry is also expected to assist drainage, as even a slight inclination would encourage residual fluid to collect at the outlet rather than pool within the sensing volume. Any thin residual film remaining on the electrode surfaces after air purging is expected to contribute minimally to the measured capacitance relative to the bulk fluid, given that the film thickness is small compared to the interelectrode gap. However, this contribution was not independently quantified and may become non-negligible for fluids with substantially higher viscosity or stronger surface adhesion. For viscous fluids such as ethylene glycol and propylene glycol, repeated measurement cycles without solvent rinsing produced a gradual upward drift in the dry air baseline capacitance, suggesting progressive surface accumulation, while fluid capacitance readings remained stable within the tested range (Figure S7). To mitigate this, a compatible solvent injection at 40 mL/min followed by solvent withdrawal and an increased-volume air purge is recommended. Acetone was used here to suppress the observed baseline drift at the cost of approximately 90 s of additional overhead per measurement cycle. Monitoring the air baseline capacitance between measurements and triggering a solvent

rinse only when it exceeds a user-defined threshold would limit the throughput penalty to cycles where accumulation is actually detected.

S3. Autonomous Ternary Phase Diagram Mapping

S3.1 Gaussian-process Classification for Binodal Curve Inference

We implemented autonomous ternary phase diagram mapping using the Gaussian Process Classifier from *scikit-learn* with a radial basis function kernel (initial length scale = 1.0, optimization bounds = 0.05 to 2.0). The kernel models smooth transitions between miscible and immiscible regions, with the length scale parameter controlling the spatial correlation decay and optimized during training via maximum likelihood estimation with two optimizer restarts. The classifier operates on the first two composition coordinates in volume fraction (f_1, f_2) with the third component determined by mass balance ($f_3 = 1 - f_1 - f_2$). Initial compositions are sampled along a diagonal scan line to identify regions containing the miscibility boundary, ensuring at least one example of each class is obtained before active learning begins. At each iteration, the classifier is retrained on all accumulated experimental labels and predicts the probability of miscibility $P(miscible|f_1, f_2)$ across a candidate grid with step size of 0.01 covering the ternary composition space, subject to a minimum separation constraint of 0.03 from any existing measurement point to prevent redundant sampling.

The active learning acquisition function selects subsequent compositions based on uncertainty sampling, prioritizing points where $P(\text{miscible})$ is closest to 0.5, indicating maximum classification uncertainty. The loop terminates when no remaining candidate compositions exhibit uncertainty above the tolerance threshold (i.e., when the most uncertain remaining point has $|P(miscible) - 0.5| > 0.45$, meaning all points have $P(miscible) < 0.05 \; or \; P(miscible) > 0.95$), or when maximum iterations (100) are reached. For visualization, the binodal curve was extracted as the $P(miscible) = 0.5$ contour using matplotlib.pyplot.tricontour and fitted via 6th-order polynomial to reduce discretization artifacts.

S3.2 Nonlinear Programming for Tie Lines Determination

We consider a ternary liquid system with components $i \in \{1,2,3\}$ in a two-phase region. For each experiment ("run") a known set of injection volumes is introduced into the chamber, and three sensor signals are measured: two dielectric quantities (capacitance C and loss D) and an

optical ratio R. The goal is, for each run, to infer the compositions of the top and bottom equilibrium phases, $\mathrm{x}^T = (x_1^T, x_2^T, x_3^T)$ and $\mathrm{x}^B = (x_1^B, x_2^B, x_3^B)$, the phase split α (mole fraction of total moles in the top phase), and the interface height, subject to overall mass balance, prior knowledge of the miscibility gap, and geometric constraints of the cell.

The overall feed composition x^F for a run is obtained from the known injection volume ratios. Given input volume ratios v_i^{inj} and a target fill volume V_{tot}, we first rescale the volumes so that $\sum_i V_i = V_{\mathrm{tot}}$ via $V_i = s \times v_i^{\mathrm{inj}}$ with $s = V_{\mathrm{tot}} / \sum_j v_j^{\mathrm{inj}}$. This rescaling ensures that the total injected volume equals the fixed column volume (7.5 mL), so the cell is fully filled while preserving the specified composition ratios. Each component has density ρ_i and molar mass M_i; the corresponding partial molar volume is approximated as $\bar{v}_i = M_i / \rho_i$. The number of moles of component i in the cell is then $n_i = V_i / \bar{v}_i$, with total moles $N_{\mathrm{tot}} = \sum_i n_i$. The overall feed mole fractions are:

$$x_i^F = \frac{n_i}{N_{\mathrm{tot}}}, \qquad where \sum_i x_i^F = 1, \tag{S7}$$

and x^F is treated as the feed point in composition space for that run.

The cell is modeled as a vertical column of total volume V_{tot}. The top portion of volume V_{elec} is the dielectric region seen by the electrodes, and a small bottom portion is where the optics effectively probe the fluid. At equilibrium the column is partitioned into a bottom phase (volume V_B) and a top phase (volume $V_T = V_{\mathrm{tot}} - V_B$). Let ρ_i^{mol} be the molar density of pure component i in mol/mL. The molar densities of the two phases are approximated by ideal composition-weighted averages:

$$\rho_T^{\mathrm{mol}} = \sum_i x_i^T \rho_i^{\mathrm{mol}} \quad and \quad \rho_B^{\mathrm{mol}} = \sum_i x_i^B \rho_i^{\mathrm{mol}} \tag{S8}$$

Given a candidate phase split $\alpha = N_T / N_{\mathrm{tot}}$, where N_T and $N_B = N_{\mathrm{tot}} - N_T$ are the moles in top and bottom phases, we obtain phase volumes:

$$V_T = \frac{\alpha N_{\mathrm{tot}}}{\rho_T^{\mathrm{mol}}} \quad and \quad V_B = \frac{(1-\alpha) N_{\mathrm{tot}}}{\rho_B^{\mathrm{mol}}} \tag{S9}$$

These are rescaled by a common factor so that $V_T + V_B = V_{\mathrm{tot}}$, which fixes the interface height $h = V_B$ in volume coordinates. The electrode region occupies the top interval of the column of length

V_{elec}, i.e. the volume range $[V_{\text{tot}} - V_{\text{elec}}, V_{\text{tot}}]$. The bottom phase occupies $[0, h]$; the top phase occupies $[h, V_{tot}]$. The volumes of each phase within the electrode region are therefore

$$V_B^{\text{elec}} = \max(0, V_{\text{elec}} - (V_{\text{tot}} - h)) \quad and \quad V_T^{\text{elec}} = V_{\text{elec}} - V_B^{\text{elec}} \tag{S10}$$

and the corresponding volume fractions seen by the dielectric sensors are $f_T = V_T^{\text{elec}}/V_{\text{elec}}$ and $f_B = 1 - f_T$. This geometric mapping, implemented via a function that converts α, x^T, and x^B into f_T, ensures that the dielectric signals are always mixed on a physically meaningful volume basis rather than directly in terms of α.

For each sensor, we construct a forward model from calibration data. Calibration runs consist of known compositions (pure components, binaries, and possibly ternaries), with measured sensor responses (C, D, R). All compositions are converted to mole fractions using the same density/molar-mass procedure as for the feed. The sensor models map composition to signal for a homogeneous phase: $C = f_C(\mathrm{x})$, $D = f_D(\mathrm{x})$, $R = f_R(\mathrm{x})$. In the implementation used here, the sensors are calibrated using the three pure components, treated as endpoints of the two relevant binary edges. A scalar composition coordinate z is defined as the mole fraction of the intermediate component within each edge (e.g. $z = x_2/(x_1 + x_2)$ or $z = x_2/(x_2 + x_3)$), and simple polynomials in z (implemented here as quadratic) are then fit and evaluated at the compositions of the top and bottom phases. The optical ratio is modeled primarily as a function of the bottom-phase composition, consistent with the optics probing a bottom-dominated region. This assumption breaks down in the rare case where the interface drops below the optical region, such that the bottom phase no longer occupies the optical sensing volume.

Given a candidate tie line $(\mathrm{x}^T, \mathrm{x}^B)$ from the miscibility gap, the phase split α is determined by enforcing the lever rule as closely as possible. We treat x^T and x^B as points in composition space and project the feed point x^F onto the line segment between them:

$$\mathrm{v} = \mathrm{x}^T - \mathrm{x}^B, \alpha = \frac{(\mathrm{x}^F - \mathrm{x}^B) \cdot \mathrm{v}}{\mathrm{v} \cdot \mathrm{v}} \tag{S11}$$

Candidates for which $\alpha \notin [0,1]$ are rejected. For acceptable α, the implied mixture composition is $\mathrm{x}^{\text{mix}} = \alpha \mathrm{x}^T + (1 - \alpha)\mathrm{x}^B$. Any residual violation of the lever rule is quantified by $\mathrm{r}_{\text{mass}} = \mathrm{x}^F - \mathrm{x}^{\text{mix}}$ and contributes a quadratic penalty to the objective.

For each run, the predicted macroscopic sensor responses are constructed by combining the homogeneous-phase models with the geometric mixing described above. For the dielectric sensors, the predicted values are:

$$C_{\text{calc}} = f_T\, f_C(\mathrm{x}^T) + f_B\, f_C(\mathrm{x}^B) \quad and \quad D_{\text{calc}} = f_T\, f_D(\mathrm{x}^T) + f_B\, f_D(\mathrm{x}^B) \tag{S12}$$

where f_T and f_B depend on α, x^T, and x^B through the molar densities and electrode geometry. The optical ratio is assumed to be dominated by the bottom slice, so the predicted value is taken as $R_{\text{calc}} = f_R(\mathrm{x}^B)$. The measured sensor values for the run are denoted C_{meas}, D_{meas}, and R_{meas}.

The miscibility gap is represented by a discrete set of tabulated compositions $\{\mathrm{x}_k^{\text{binodal}}\}$ (the binodal curve) ordered along the curve. We identify the plait point index k^* as the index at which the mole fraction of the intermediate component reaches its maximum. This index partitions the binodal into two branches: an "aqueous-like" branch $\{\mathrm{x}_k^{\text{binodal}}: k \leq k^*\}$ that connects the plait point to one pure-component vertex, and an "organic-like" branch $\{\mathrm{x}_k^{\text{binodal}}: k \geq k^*\}$ that connects the plait point to the other vertex. The plait point belongs to both branches, ensuring that all tie lines rich in the intermediate component remain accessible. In the search, top-phase candidates are taken from the organic-like branch and bottom-phase candidates from the aqueous-like branch.

For a single run, we define a total objective function

$$J = J_{\text{mass}} + J_{\text{sens}} + J_{\text{phys}} \tag{S13}$$

with three contributions. The mass-balance term enforces straight tie lines in composition space,

$$J_{\text{mass}} = \frac{1}{\sigma_{\text{mass}}^2} \parallel \mathrm{r}_{\text{mass}} \parallel_2^2 \tag{S14}$$

where σ_{mass} is a small tolerance for deviations from the lever rule. The sensor-matching term penalizes discrepancies between predicted and measured sensor values,

$$J_{\text{sens}} = w_C \left(\frac{C_{\text{calc}} - C_{\text{meas}}}{\sigma_C}\right)^2 + w_D \left(\frac{D_{\text{calc}} - D_{\text{meas}}}{\sigma_D}\right)^2 + w_R \left(\frac{R_{\text{calc}} - R_{\text{meas}}}{\sigma_R}\right)^2 \tag{S15}$$

with user-selected weights w_C, w_D, w_R and scale parameters $\sigma_C, \sigma_D, \sigma_R$ reflecting relative sensor uncertainties and trust.

The term J_{phys} encodes an adaptive immiscibility prior that regularizes the inverse problem by favoring phase compositions consistent with established physical expectations for the ternary system. While the optimization can, in principle, be performed without such a prior, we find that incorporating physically informed constraints substantially improves robustness, suppresses spurious tie lines, and accelerates convergence toward physically meaningful solutions. To avoid over-constraining the inversion, the prior is formulated adaptively and relaxes automatically when required by the data. Prior information typically includes expectations that certain components are present only at very low mole fraction in one of the phases (e.g., a component expected to be minor in the bottom phase). We define a raw immiscibility penalty $p(\mathrm{x}^T, \mathrm{x}^B)$ that accumulates squared excesses above phase-specific thresholds. For example, if component 1 is expected to be nearly absent from the bottom phase and component 3 from the top phase, typical terms would take the form:

$$p(\mathrm{x}^T, \mathrm{x}^B) = \left[\frac{\max\left(0, x_3^T - \theta_3^T\right)}{\delta_3^T}\right]^2 + \left[\frac{\max\left(0, x_1^B - \theta_1^B\right)}{\delta_1^B}\right]^2 + \cdots \qquad \text{(S16)}$$

where θ_i^{phase} are threshold mole fractions and δ_i^{phase} are soft tolerances. In practice, the code uses a small number of such terms corresponding to the components known a priori to be minor in each phase. The physical prior contribution to the objective is then:

$$J_{\text{phys}} = w_{\text{phys}}^{(\ell)}\, p_{\theta^{(\ell)}}(\mathrm{x}^T, \mathrm{x}^B) \qquad \text{(S17)}$$

where both the prior weight $w_{\text{phys}}^{(\ell)}$ and the thresholds $\theta^{(\ell)}$ depend on a discrete relaxation level ℓ.

To avoid over-constraining the inversion while still exploiting prior knowledge, we implement a multi-level adaptive relaxation scheme. Four relaxation levels $\ell = 0,1,2,3$ are defined: level 0 ("strict") uses a large prior weight and tight thresholds (strongly penalizing even modest violations), level 1 ("relaxed") uses a smaller weight and looser thresholds, level 2 ("very relaxed") loosens them further, and level 3 ("no priors") sets the physical prior weight to zero, effectively disabling immiscibility penalties. For each run, the algorithm proceeds as follows. We start at level $\ell = 0$ and, for that level's configuration $\left(w_{\text{phys}}^{(\ell)}, \theta^{(\ell)}\right)$, exhaustively search over all permissible pairs $(\mathrm{x}^T, \mathrm{x}^B)$ on the two binodal branches (subject to additional global constraints such as non-reuse of binodal points across runs). For each candidate pair we compute α, reject if $\alpha \notin [0,1]$, and evaluate the objective J. The best candidate at level ℓ is characterized by its minimal

J and mass-residual norm $\| r_{\text{mass}} \|$. If this candidate satisfies both $J < J_{\text{th}}$ and $\| r_{\text{mass}} \| < \Delta_{\text{mass}}$ (with prescribed thresholds J_{th} and Δ_{mass}), it is accepted as the solution for that run. If either criterion fails and $\ell < 3$, the algorithm increases the relaxation level to $\ell + 1$, thereby weakening or disabling the immiscibility prior, and repeats the search. If all levels are exhausted, the best available candidate at the highest level is retained. In this way, the solver enforces a strong immiscibility prior whenever it is compatible with the data, but automatically relaxes towards a more data-driven mode when necessary, rather than forcing the inversion to fit unrealistic phase compositions or severely bending tie lines.

Across multiple runs, feed compositions are computed from the injection volumes and sorted by increasing co-solvent mole fraction to encourage a smooth progression along the binodal. For each run, the adaptive search yields x^T, x^B, α, the electrode volume fraction f_T, predicted sensor responses, the mass residual norm, and the relaxation level ultimately required, providing a framework for inferring ternary tie lines from combined capacitance and optical measurements without requiring direct sampling or analytical characterization of the separated phases.

3.2.1 Effect of Excess Molar Volume on Phase-volume and Interface Height Mapping

The phase molar densities in Equation S8 are approximated from pure-component density information and do not explicitly include the excess molar volume, V^E, of each liquid phase. This approximation affects the conversion from candidate phase compositions and phase split to phase volumes, interface height, and electrode-region volume fractions. A more general molar volume expression for phase p, where $p = T$ or B, is

$$\bar{V}_p(x_p, T) = \sum_i x_{i,p} \, \bar{V}_i^0(T) + V_p^E(x_p, T) \tag{S18}$$

where $\bar{V}_i^0 = M_i/\rho_i$ is the pure-component molar volume. The corresponding corrected phase molar density is

$$c_p^{\text{corr}}(x_p, T) = \frac{1}{\bar{V}_p(x_p, T)} \tag{S19}$$

Thus, the current Equation S8 phase-density estimate can be replaced by c_T^{corr} and c_B^{corr} before applying Equation S9. This correction does not otherwise change the nonlinear programming framework, because the lever-rule projection, sensor-response prediction, and binodal constraints can be evaluated using the same workflow once the corrected phase molar densities are supplied.

The sensitivity of the normalized bottom-phase volume fraction,

$$f_B = \frac{V_B}{V_T + V_B} \tag{S20}$$

to excess volume can be estimated analytically. Defining the fractional excess volume of phase p as

$$\epsilon_p = \frac{V_p^E}{\sum_i x_{i,p} \bar{V}_i^0} \tag{S21}$$

a first-order expansion gives

$$\Delta f_B \approx f_B(1 - f_B)(\epsilon_B - \epsilon_T) \tag{S22}$$

This expression shows that two factors limit the magnitude of the interface-height error. First, the phase volumes V_T and $V_B$are renormalized to the fixed chamber volume, so a contraction common to both phases has a negligible effect on the normalized interface position; only the differential contraction, $\epsilon_B - \epsilon_T$, enters to first order. Second, the geometric factor $f_B(1 - f_B)$ reaches its maximum value of 0.25 only when the interface is at mid-height, $f_B = 0.5$. Therefore, even a differential fractional contraction of 4% would give a maximum normalized phase-volume error of approximately

$$|\ \Delta f_B\ |_{\max} \approx 0.25 \times 0.04 = 0.01 \tag{S23}$$

For the ethyl alcohol/hexane/water validation system, the coexisting phases are an organic-rich top phase and an aqueous-rich bottom phase. The strongest excess-volume contribution is expected from the ethyl alcohol/water interaction. Literature data for water/ethanol mixtures at 298.15 K show negative excess molar volumes, with a minimum of approximately $-1.07\ \mathrm{cm}^3/\mathrm{mo}l$ near intermediate composition.[6] Based on the ideal molar volume at this composition, this corresponds to approximately 3–4% fractional contraction. Because the equilibrium phases in the ethyl alcohol/hexane/water system are not both near this maximum-contraction water/ethanol composition, we use 4% as a conservative upper bound for $|\ \epsilon_B - \epsilon_T\ |$ in the present system. Using this conservative bound gives $|\ \Delta f_B\ |_{\max} \approx 0.01$, corresponding to approximately 0.075 mL or 0.75 mm in the 7.5 mL, 75 mm chamber. This scale is small relative to the electrode-region volume and is consistent with the observed tie-line average absolute error

of 0.01 mol fraction for the ethyl alcohol/hexane/water validation system. Therefore, excess-volume effects are not expected to be a dominant source of error in the tested system.

The constrained NLP formulation further limits, but does not eliminate, propagation of small phase-volume errors. Candidate solutions are restricted to the experimentally inferred binodal branches and must simultaneously satisfy phase-split bounds, lever-rule consistency, sensor-response agreement, and adaptive physical priors. Therefore, a small error in the predicted interface height does not translate into an unconstrained change in extracted phase compositions. Instead, the optimizer identifies the top- and bottom-phase compositions that best satisfy all constraints simultaneously. However, this should not be interpreted as an automatic correction for excess molar volume: the predicted interface height is still generated through the phase-volume model in Equations S8–S10.

For future applications to systems with larger or more asymmetric excess molar volumes, explicit density corrections should be incorporated. This can be done by replacing the current Equation S8 phase-density estimate with composition-dependent molar densities obtained from measured mixture-density data, Redlich–Kister-type excess-volume correlations, or other volumetric models.

S3.3 Error Metric (Average Absolute Error)

To quantify the accuracy of platform measurements against literature values for both ternary binodal curves and tie lines, we calculated the average absolute error (AAE). For ternary systems, compositions are represented as (x_1, x_2, x_3) for components 1, 2, and 3, where x_i denotes volume fraction for binodal curves and mole fraction for tie lines. The absolute error for each component measurement is:

$$\text{Error}_i = | x_i^{\text{lit}} - x_i^{\text{exp}} | \quad \text{(S24)}$$

where i denotes the component (1, 2, or 3).

The AAE across all measurements is:

$$\text{AAE} = \frac{1}{N} \sum_{k=1}^{N} \text{Error}_k \quad \text{(S25)}$$

where N is the total number of individual component measurements. For binodal curves, N equals the number of experimental points multiplied by 3 components. For tie lines, N equals the number of tie lines multiplied by 2 phases (top and bottom) multiplied by 3 components. Binodal mapping accuracy was evaluated on a volume fraction basis, while tie line determination accuracy was evaluated on a mole fraction basis to match what was measured and obtained from the platform.

S3.4 Reverse-mapping Capability for Formulation Adjustment

To support targeted formulation work and experiment planning, the trained GPC was utilized to perform reverse-mapping calculations. This algorithm determines the exact quantity of a single pure component required to transition an arbitrary starting mixture across the binodal curve, shifting it from a one-phase to a two-phase state, or vice versa.

Geometrically, the addition of a pure component to an existing mixture shifts the system's overall composition along a straight "dilution line" originating from the initial mixture's coordinates and terminating at the respective pure component's vertex on the ternary diagram. To calculate the precise intersection of this path with the phase boundary, the dilution line is parameterized by a fractional distance, $\mathrm{t} \in [0,\ 1]$, where $t = 0$ represents the initial mixture and $t = 1$ represents the pure component. The composition along this path in transformed Cartesian space $(x(t), y(t))$ is given by:

$$x(t) = (1 - t)x_0 + tx_v \quad and \quad y(t) = (1 - t)y_0 + ty_v \tag{S26}$$

where (x_0, y_0) is the initial mixture and (x_v, y_v) is the target pure component vertex. The binodal boundary is defined as the contour where the GPC predicts a phase probability of 0.5. By evaluating the GPC probability at the endpoints of the line, the algorithm determines if the path crosses the phase boundary. If a crossing occurs, Brent's method (a root-finding algorithm) is employed to calculate the exact fractional distance, t_{cross}, where the probability equals 0.5. Once t_{cross} is determined, the required amount of the pure component to add, ΔV, relative to the initial mass or volume of the mixture V_0, is calculated using the geometric ratio:

$$\Delta V = V_0 \times \frac{t_{cross}}{1 - t_{cross}} \tag{S27}$$

The final composition at the phase boundary is calculated as the weighted sum of the initial mixture and the added pure component at t_{cross}. Because the algorithm evaluates the trajectory toward

each vertex independently, the outputs represent mutually exclusive formulation pathways. This provides the user with distinct, alternative options (e.g., adding pure component 1, component 2, or component 3) to achieve the desired phase transition.

Supplementary Figures

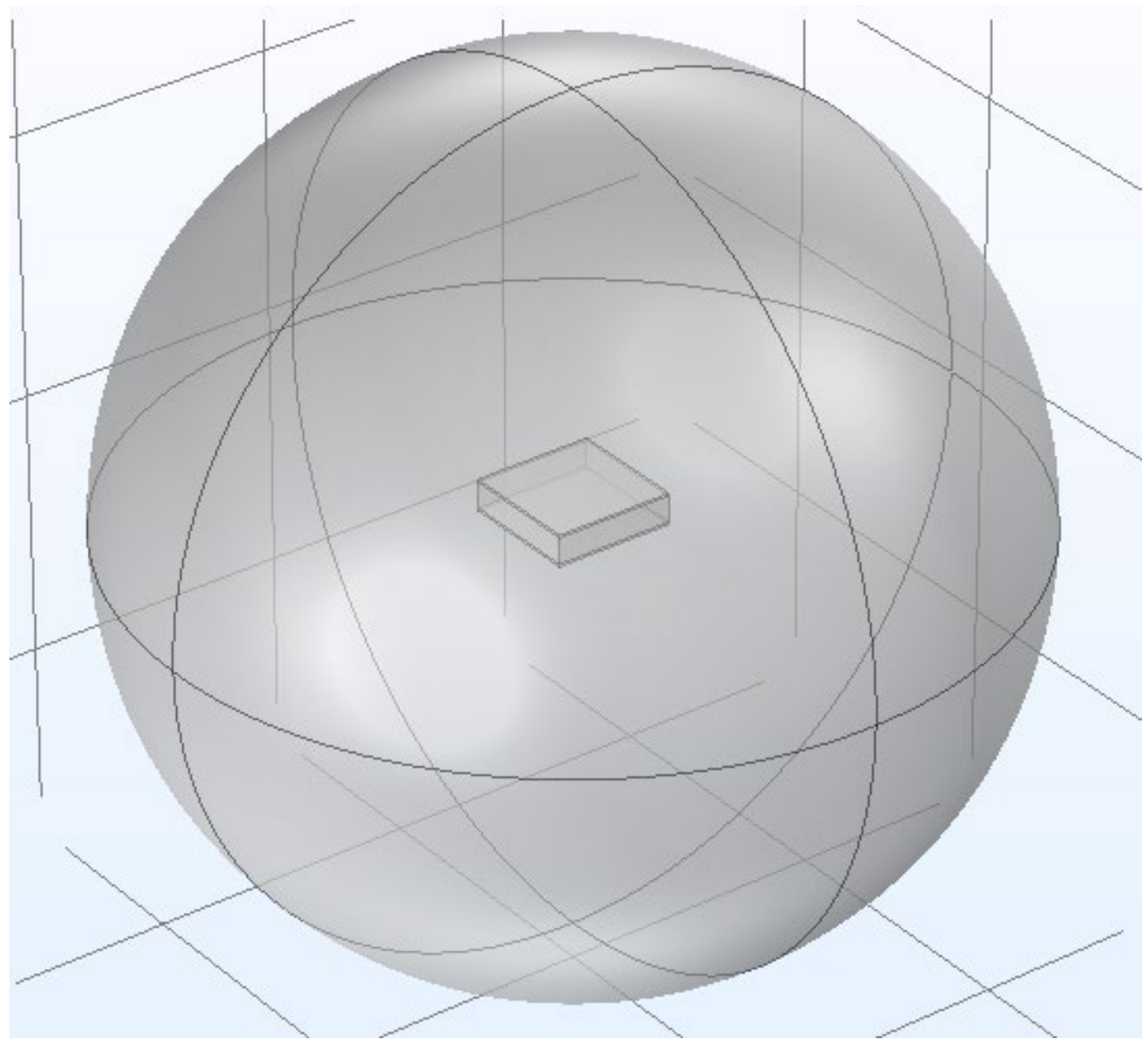

Figure S1. COMSOL simulation geometry of the parallel-plate capacitor enclosed in a spherical air domain.

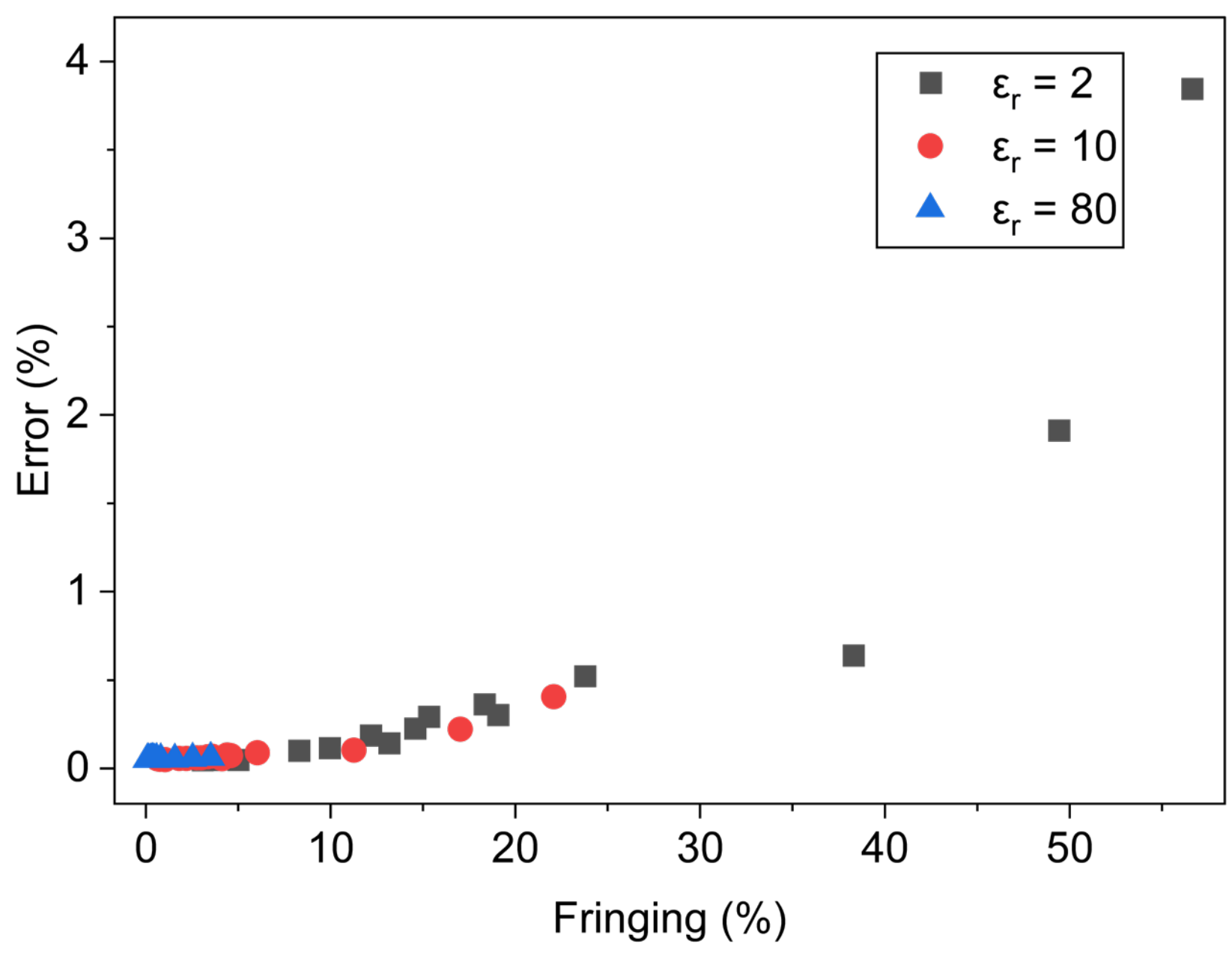

Figure S2. Fringing field percentage versus dielectric constant extraction error from COMSOL simulations at three assigned fluid dielectric constants (ε_r = 2, 10, 80).

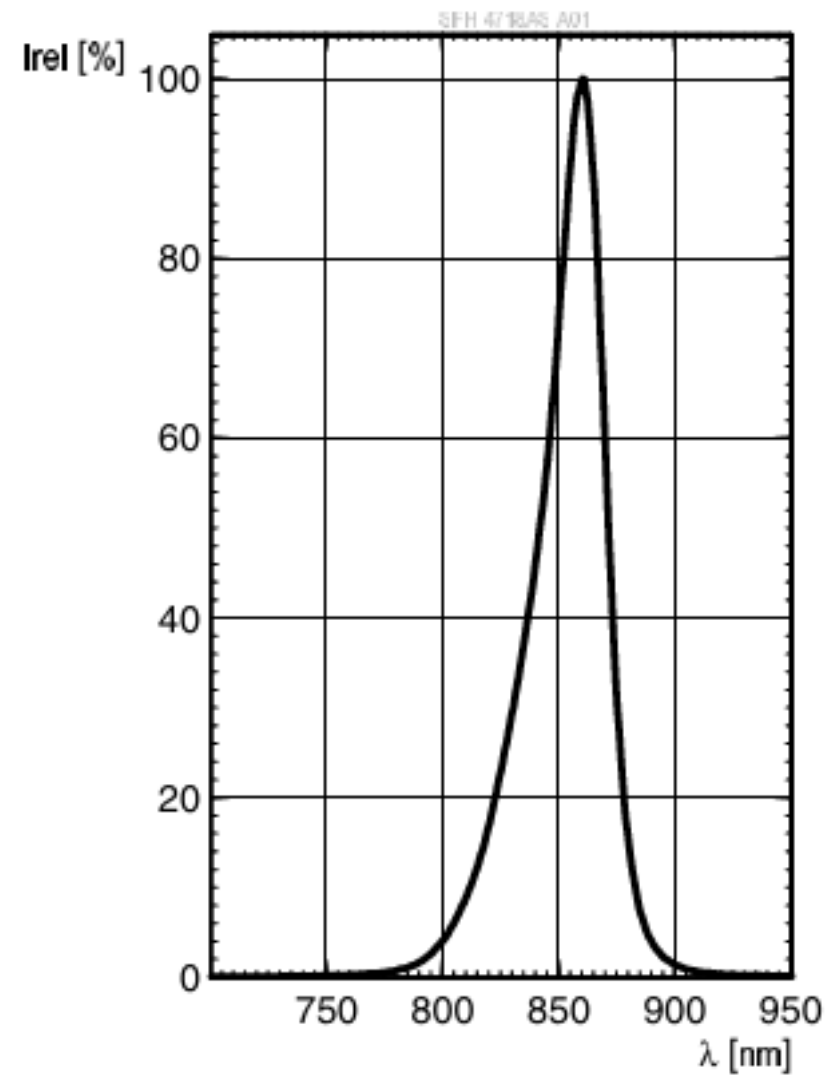

Figure S3. Relative emitted light intensity, I_{rel}, for the SFH-4718AS-A01 emitter as a function of wavelength [2].

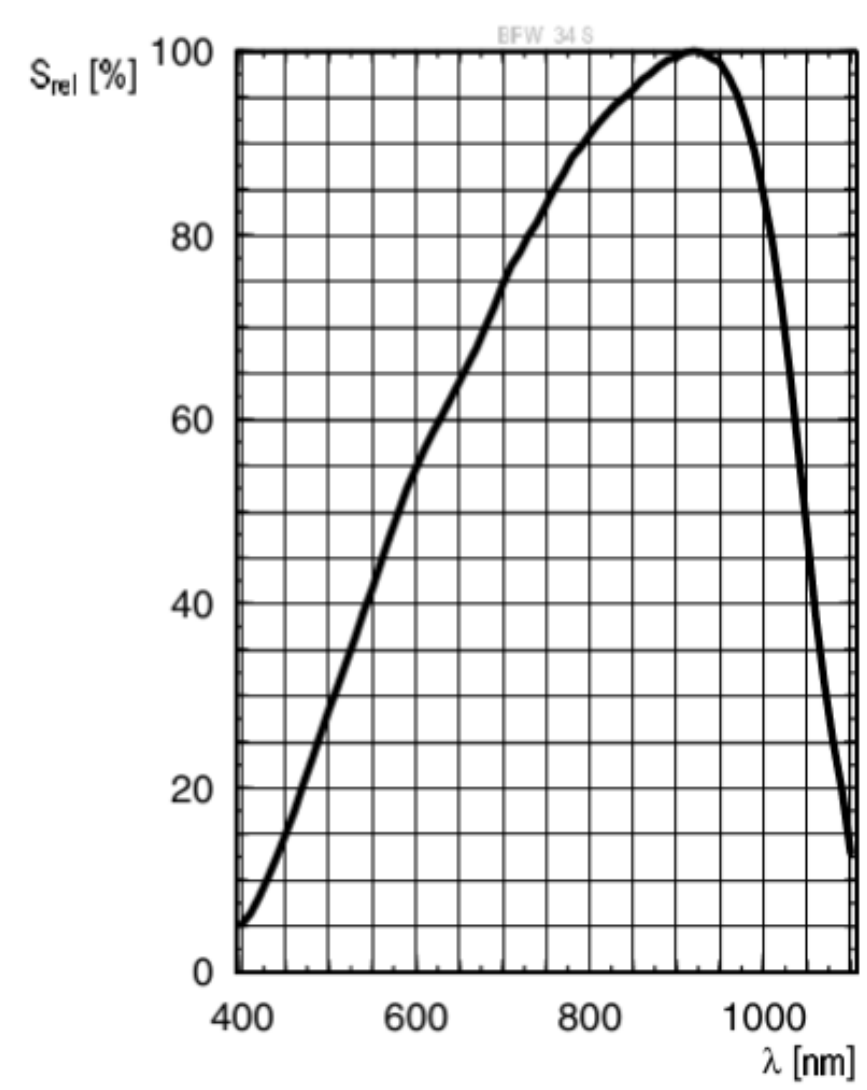

Figure S4. Relative spectral sensitivity, S_{rel}, for the BPW 34 S-Z photodiode as a function of wavelength [3].

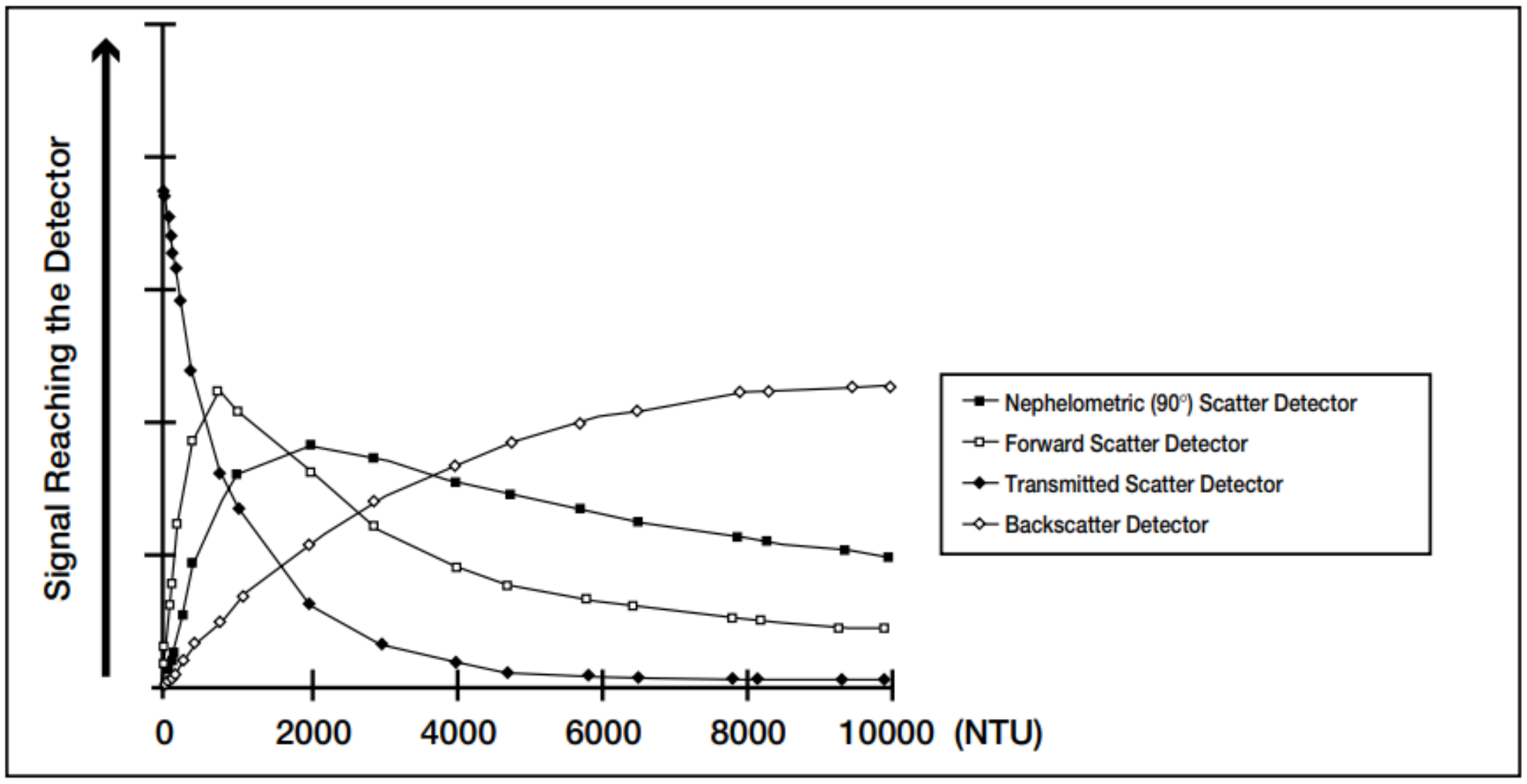

Figure S5. Turbidity response of the Hach reference instrument for scattering and transmission measurements.[7]

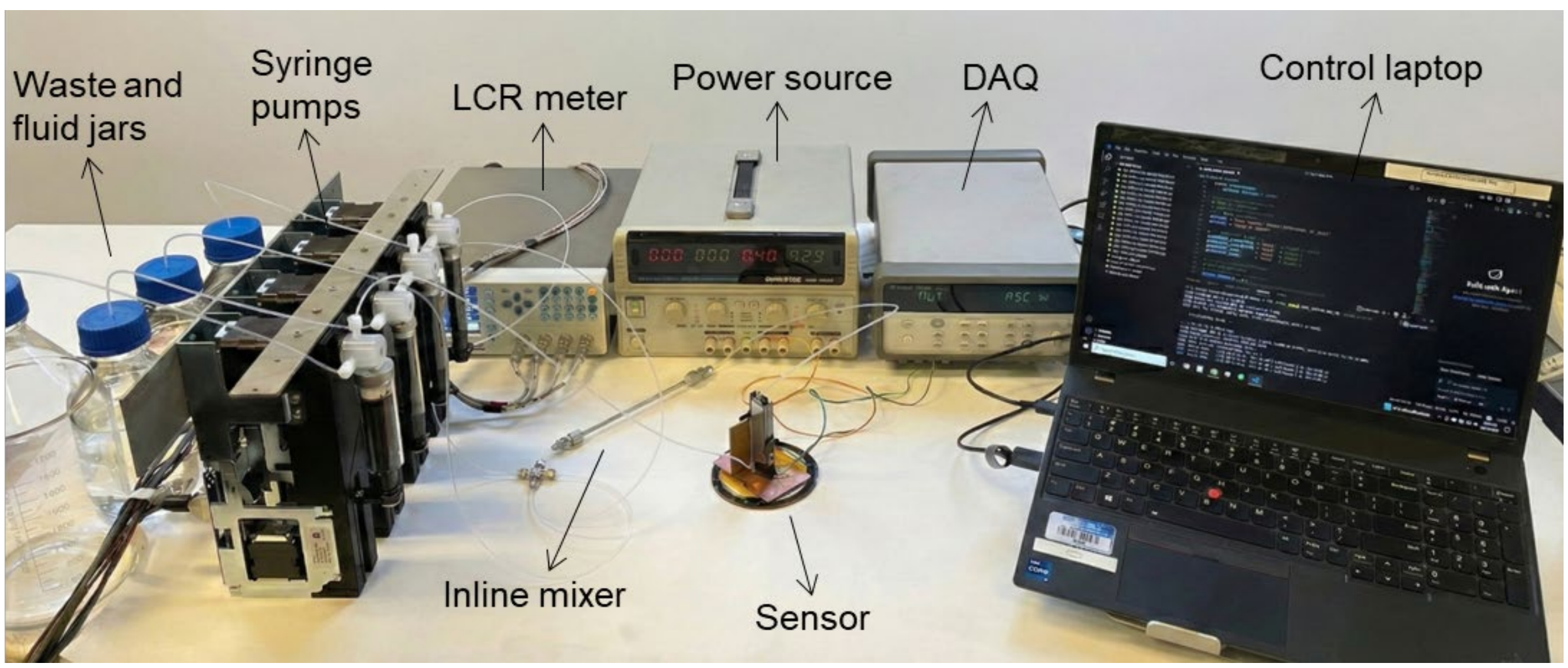

Figure S6. Automated platform components comprising multi-channel syringe pumps, waste and fluid reservoirs, the multimodal device, LCR meter, data-acquisition unit, power source, and laptop. (Background removed for clarity)

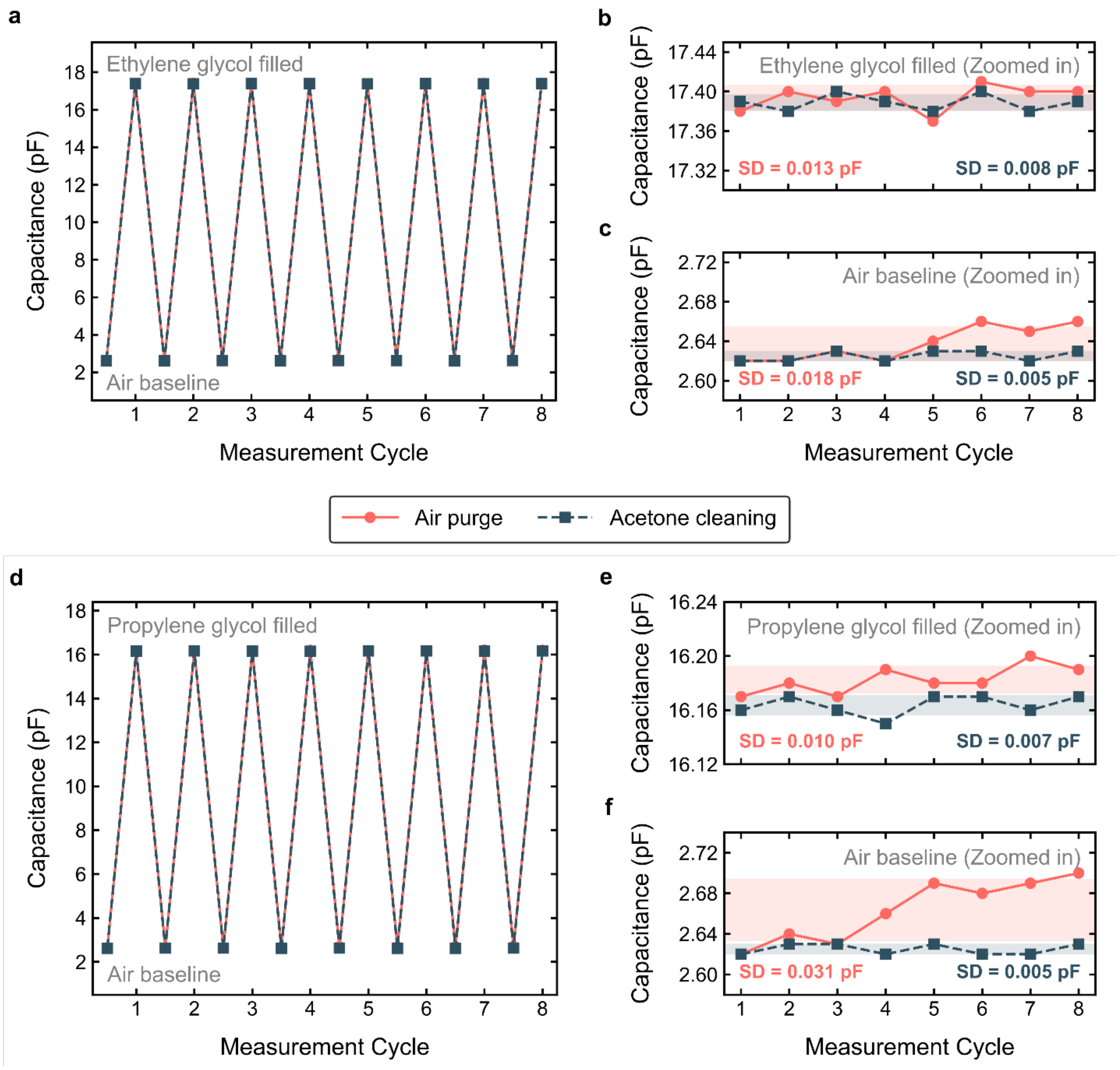

Figure S7. Capacitance measurements over eight consecutive measurement cycles for ethylene glycol (a–c) and propylene glycol (d–f) under air purge and acetone cleaning protocols. Full-range plots (a, d) show the alternating capacitance signal between fluid-filled and air baseline states for both cleaning methods. Zoomed views of the fluid-filled readings (b, e) and air baseline readings (c, f) reveal that acetone cleaning yields a lower standard deviation (SD) in the air baseline (SD = 0.005 pF for both fluids) compared to air purge alone (SD = 0.018 pF for ethylene glycol; SD = 0.031 pF for propylene glycol), consistent with progressive surface accumulation under air-purge-only conditions. Fluid capacitance readings remain comparable between the two protocols in both cases.

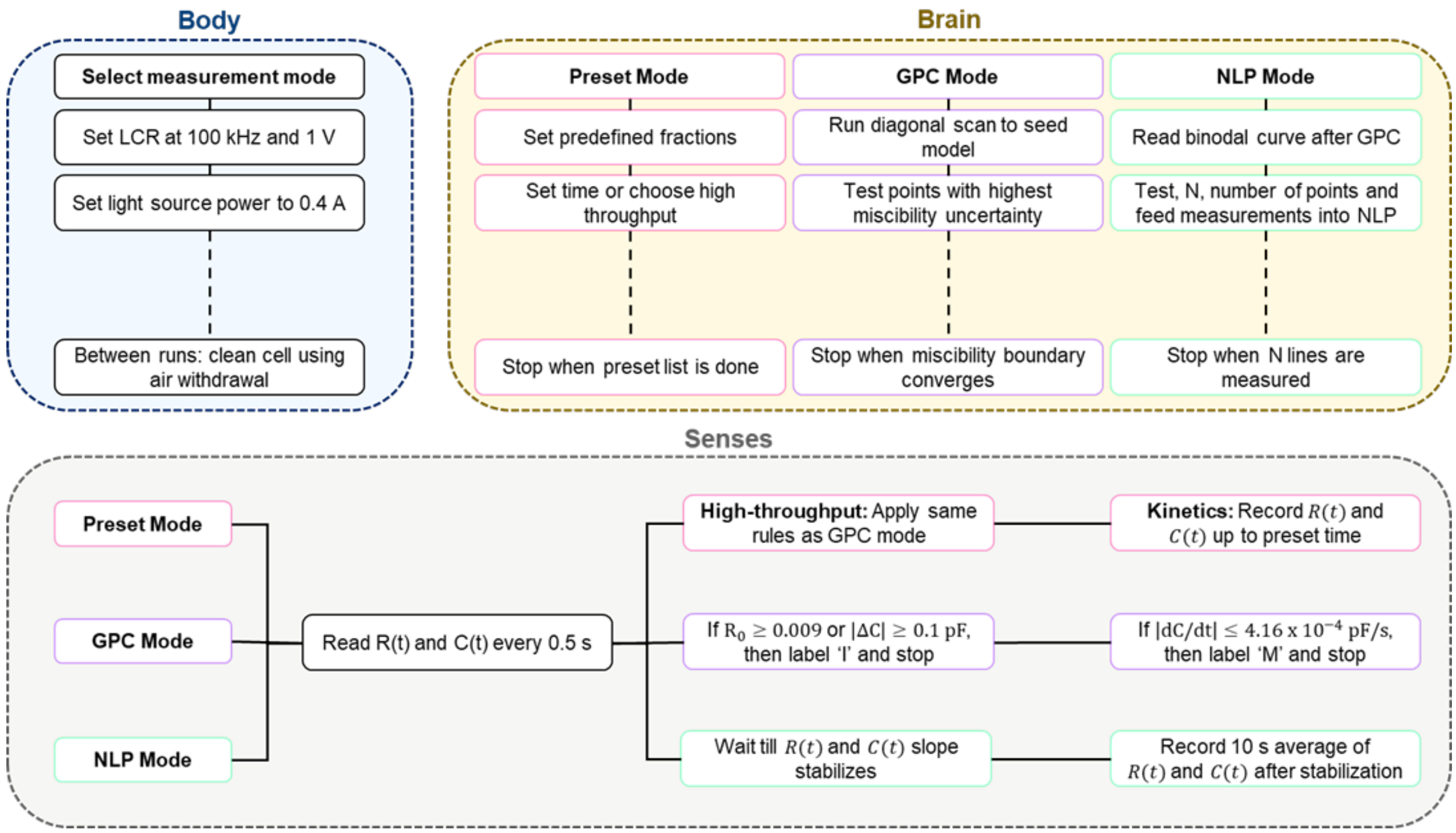

Figure S8. Measurement workflow separating hardware control ("Body"), decision-making algorithms ("Brain"), and real-time signal processing ("Senses"). The workflow operates in three modes: preset for predefined sequences, Gaussian-process classification (GPC) for autonomous ternary phase diagram mapping, and nonlinear programming (NLP) for tie line determination.

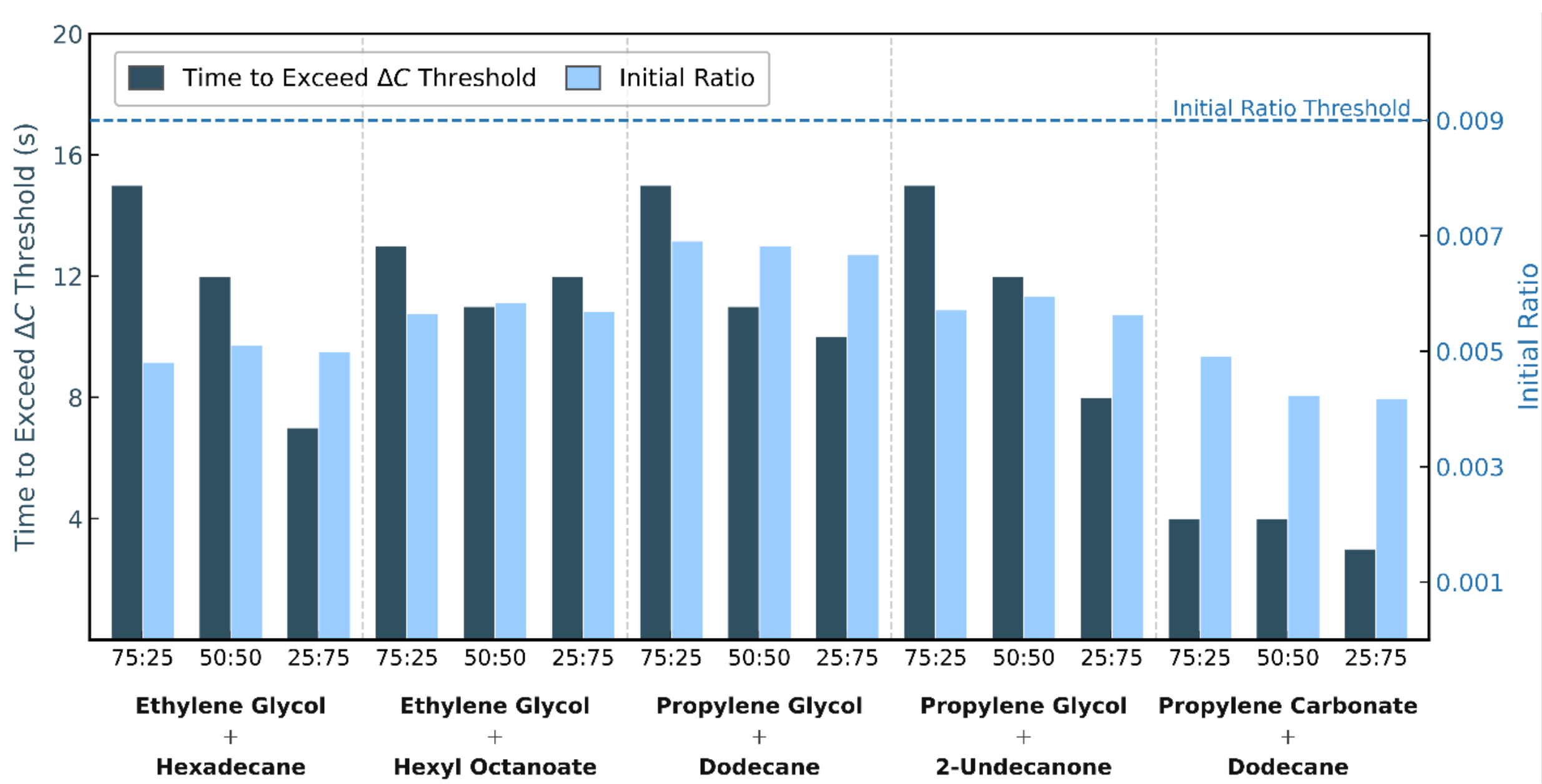

Figure S9. Time to exceed the ΔC threshold and initial optical ratio for additional isorefractive binary mixtures tested across three volume ratio compositions (75:25, 50:50, 25:75). All mixtures exceeded the ΔC threshold, confirming immiscibility, while all initial optical ratios remained below the optical threshold of 0.009 (right axis, dashed line), confirming failed optical detection across all systems and compositions tested.

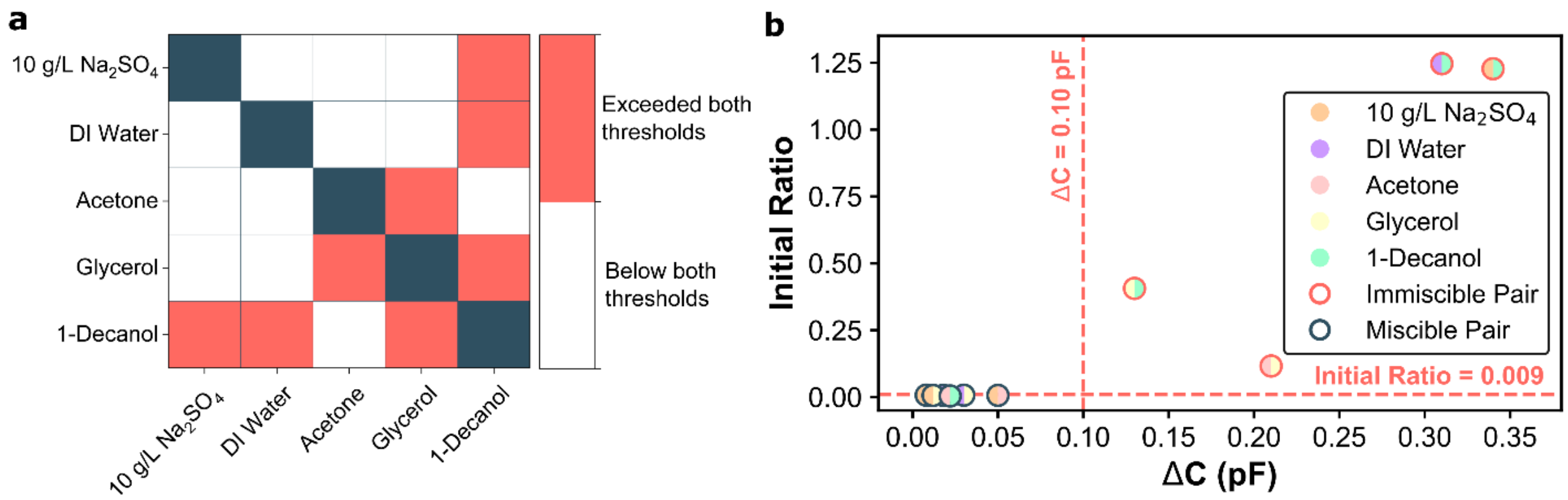

Figure S10. Miscibility classification for an extended set of fluid pairs spanning challenging chemical classes, including a saline solution (10 g/L Na_2SO_4), a volatile solvent (acetone), and a highly viscous fluid (glycerol; introduced manually, as its viscosity exceeds the operating range of the syringe pumps used in this work). (a) Classification matrix based on combined optical ratio and post-mixing capacitance deviation thresholds, where red indicates pairs exceeding both thresholds (immiscible) and white entries indicate pairs remaining below both thresholds (miscible). (b) Scatter plot of initial optical ratio versus capacitance deviation after 30s for all tested pairs, with dashed lines indicating the classification thresholds (ΔC = 0.10 pF and initial ratio = 0.009). All classifications are consistent with literature-reported miscibility for the corresponding fluid pairs.[8–10]

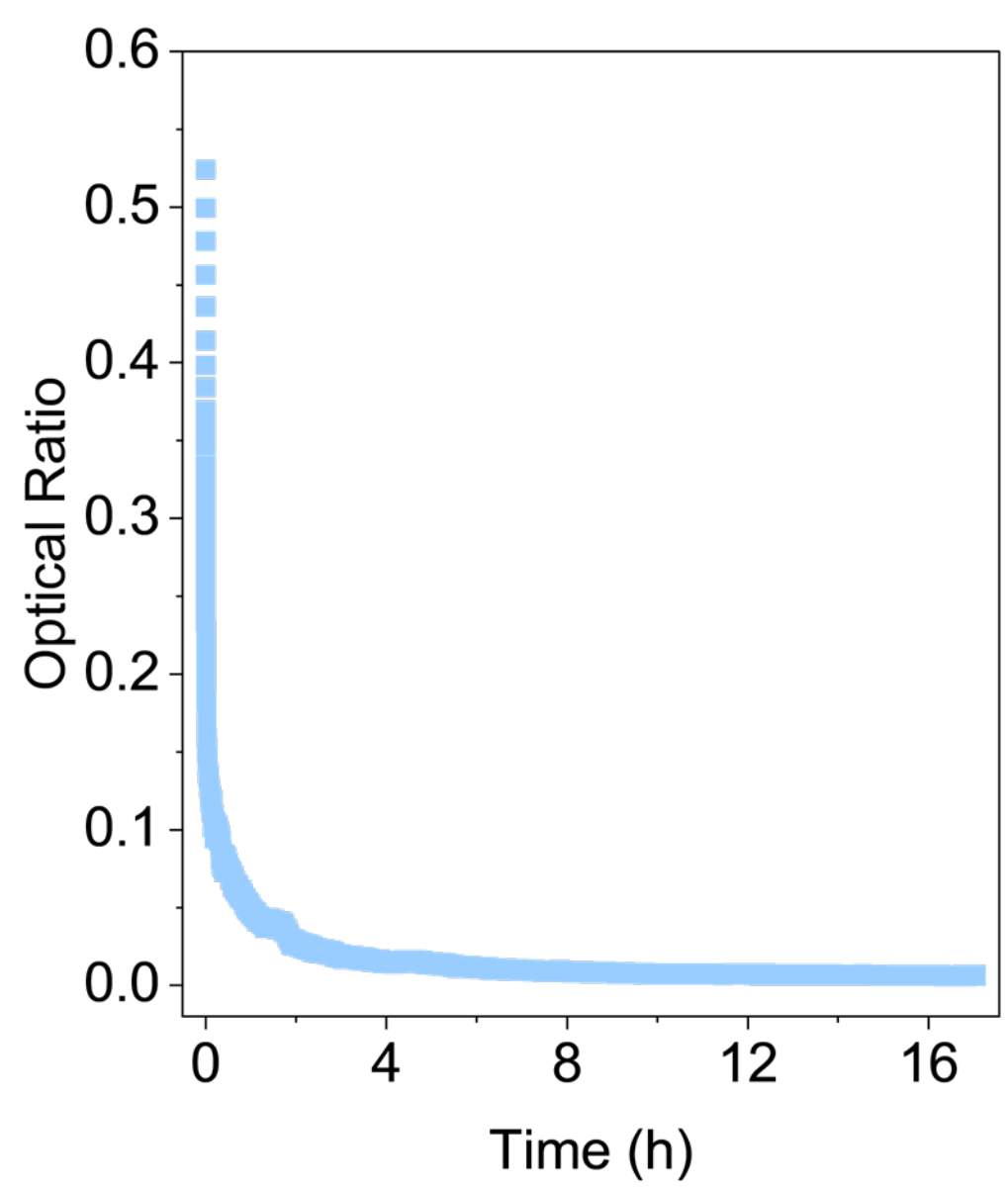

Figure S11. Optical ratio measurements recorded over 17 hours for a water/1-butanol mixture at a 30/70 volume ratio.

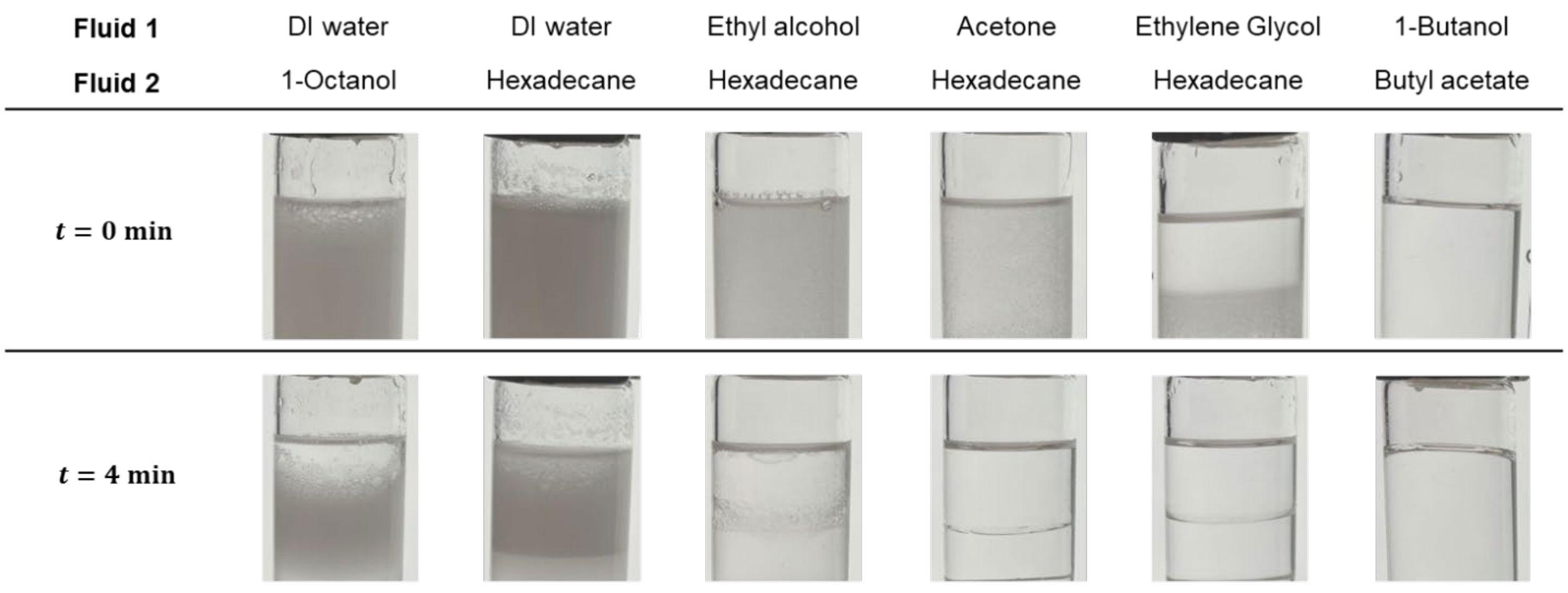

Figure S12. Visual comparison of six representative fluid mixtures immediately after mixing ($t = 0\ min$) and after 4 minutes.

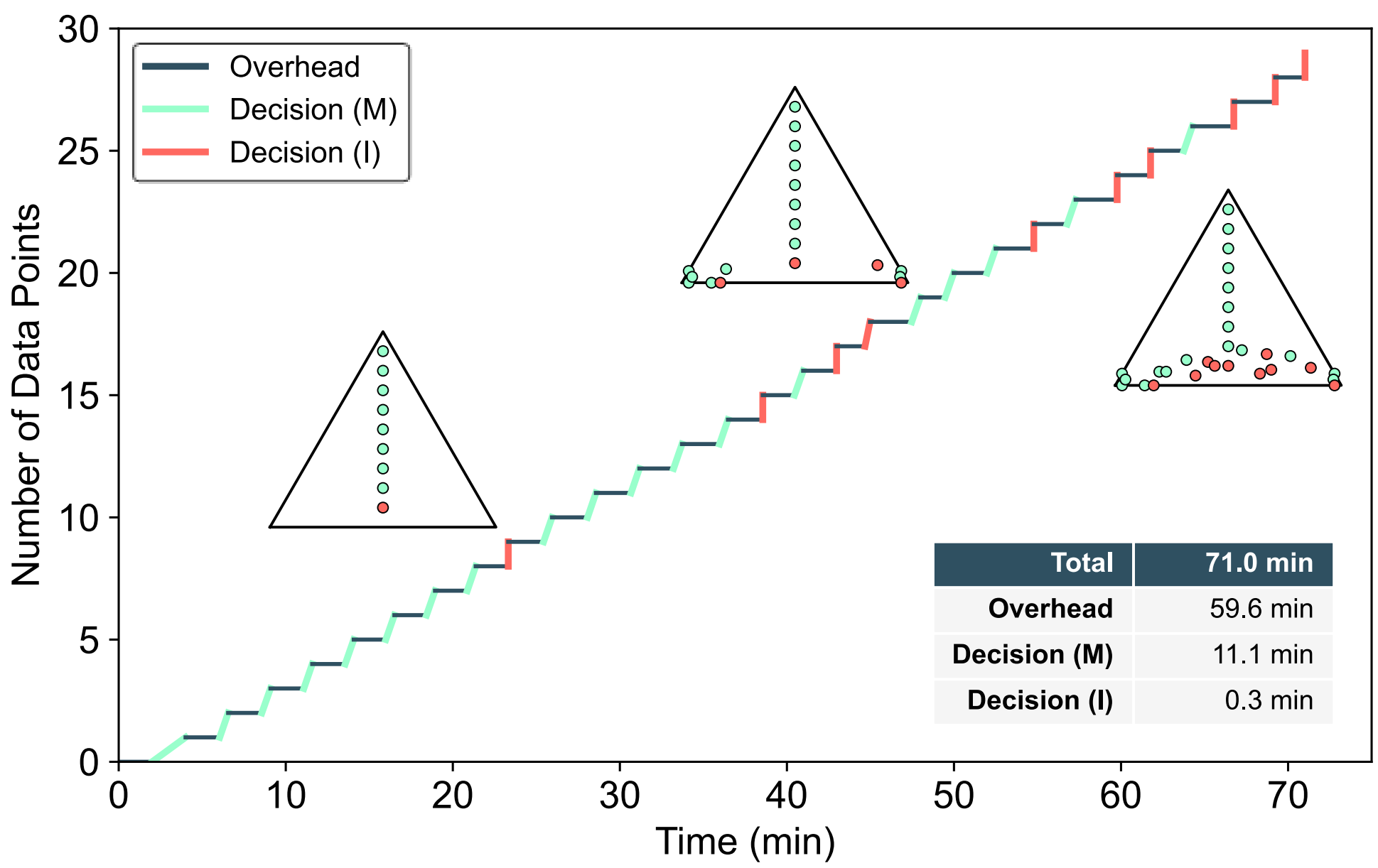

Figure S13. Time-resolved binodal acquisition for the ethyl alcohol/1-butanol/hexadecane system showing cumulative mapped points and time allocation, where overhead corresponds to pumping and withdrawal steps.

Supplementary Tables

Table S1. Dielectric constants and refractive indices for solvents used, sourced from the DIPPR Project 801.[11]

**Fluid**	**Chemical Formula**	**Dielectric Constant**	**Refractive Index**
Heptane	C_7H_{16}	1.92	1.39
Hexadecane	$C_{16}H_{34}$	2.05	1.43
Butyl Acetate	$C_6H_{12}O_2$	5.07	1.39
Acetone	C_3H_6O	20.70	1.36
1-Octanol	$C_8H_{18}O$	10.30	1.43
1-Butanol	$C_4H_{10}O$	17.84	1.40
Ethyl Alcohol	C_2H_6O	25.30	1.36
Ethylene Glycol	$C_2H_6O_2$	39.84	1.43
Water	H_2O	80.10	1.33

Table S2. Volume fractions (v_f) of ethyl alcohol, 1-butanol, and water for literature equilibrium data points and their average absolute error (AAE) relative to the fitted binodal curve.[12]

**Ethyl Alcohol v_f**	**1-Butanol v_f**	**Water v_f**	**AAE**
0.056	0.743	0.201	0.001
0.090	0.674	0.236	0.001
0.115	0.616	0.269	0.003
0.134	0.562	0.304	0.008
0.147	0.512	0.342	0.012
0.152	0.468	0.380	0.018
0.157	0.423	0.420	0.022
0.156	0.381	0.463	0.026
0.155	0.336	0.508	0.025
0.155	0.292	0.553	0.021
0.152	0.237	0.612	0.013
0.143	0.202	0.656	0.010
0.135	0.154	0.711	0.001
0.105	0.107	0.787	0.005
0.058	0.092	0.850	0.003
		**Average**	**0.011**

References

[1] X. Chen, Z. Zhang, S. Yu, T.-G. Zsurzsan, “Fringing Effect Analysis of Parallel Plate Capacitors for Capacitive Power Transfer Application,” in *Proceedings of 4th IEEE International Future Energy Electronics Conference*, IEEE, 2019.

[2] “SFH 4718AS A01 Datasheet,” *OSRAM* (2024).

[3] “BPW 34 S Datasheet,” *OSRAM* (2021).

[4] “34970A Data Acquisition/ Switch Unit Family Technical Overview,” *Keysight Technologies* (2020).

[5] “High Precision LCR Meter User Manual (MCR-6100A - MCR-6200A - MCR-6600A),” *Matrix Technology Inc.* (n.d.).

[6] J.-P. E. Grolier, E. Wilhelm, “Excess volumes and excess heat capacities of water + ethanol at 298.15 K,” *Fluid Phase Equilibria* 6 , no. 3–4 (1981) : 283–287, https://doi.org/10.1016/0378-3812(81)85011-X.

[7] M. J. Sadar, “Turbidity Science,” *Hach Company Technical Information Series Booklet No. 11* (1998).

[8] M. L. Huber, “CRC Handbook of Chemistry and Physics, 105th Edition,” *CRC Press* (2024).

[9] Z. V. Simić, I. R. Radović, M. Lj. Kijevčanin, “Measurement and Correlation of Liquid–Liquid Equilibrium Data for Ternary Systems Water + Acetone + Organic Solvents (Isoamyl Acetate, or Ethyl Butyrate, or 1-Octanol, or 1-Decanol) at 298.15 K and Atmospheric Pressure,” *Journal of Chemical & Engineering Data* 70 , no. 8 (2025) : 3283–3295, https://doi.org/10.1021/acs.jced.5c00147.

[10] S. E. Friberg, P. Liang, “Phase equilibria and structures in the system glycerol, sodium dodecyl sulfate and decanol,” *Colloid & Polymer Science* 264 , no. 5 (1986) : 449–453, https://doi.org/10.1007/BF01419550.

[11] Design Institute for Physical Properties, “DIPPR Project 801 - Full Version,” *Design Institute for Physical Property Research/AIChE* (2024).

[12] F. Ruiz, D. Prats, V. Gomis, “Quaternary liquid-liquid equilibrium. Water-ethanol-1-butanol-chloroform at 25.degree.C. Experimental determination and graphical

representation of equilibrium data," *Journal of Chemical & Engineering Data* 29 , no. 2 (1984) : 147–151, https://doi.org/10.1021/je00036a015.
